# Determinants of empiric combination antibiotic therapy for hospital associated bloodstream infections in the intensive care unit

**DOI:** 10.1038/s41598-025-22687-8

**Published:** 2025-10-20

**Authors:** Evaldas Kauzonas, Gustav Torisson, Juan Merlo, Raquel Perez, Alexis Tabah, Niccolò Buetti, Stéphane Ruckly, François Barbier, Jean-François Timsit, Fredrik Sjövall

**Affiliations:** 1 Department of Clinical Sciences, Faculty of Medicine, Lund University, Sölvegatan 19, 221 84 Lund, Sweden; 2 Department of Intensive and Perioperative Care, Skåne University Hospital Malmö, Inga Marie Nilssons Gata 47, 214 28 Malmö, Sweden; 3 Clinical Infection Medicine, Department of Translational Medicine Malmö, Lund University, Ruth Lundskogs Gata 3, 214 28 Malmö, Sweden; 4 Department of Infectious Diseases, Skåne University Hospital Malmö, Inga Marie Nilssons Gata 47, 214 28 Malmö, Sweden; 5 Unit for Social Epidemiology, Faculty of Medicine, Lund University, Jan Waldenströms Gata 35, 205 02 Malmö, Sweden; 6 Centre for Primary Health Care Research, Region Skåne, Jan Waldenströms Gata 35, 205 02 Malmö, Sweden; 7 Department of Translational Medicine-Hand Surgery, Lund University, Skåne University Hospital, Jan Waldenströms Gata 5, 205 02 Malmö, Sweden; 8 Intensive Care Unit, Redcliffe Hospital, Metro North Hospital and Health Services, Redcliffe, QLD Australia; 9 Queensland University of Technology, Brisbane, QLD Australia; 10 Faculty of Medicine, The University of Queensland, Brisbane, QLD Australia; 11 Infection Control Program and WHO Collaborating Centre, Geneva University Hospitals and Faculty of Medicine, Rue Gabrielle-Perret-Gentil 4, 1205 Geneva, Switzerland; 12 Université de Paris, INSERM, IAME UMR 1137, 75018 Paris, France; 13 ICUREsearch, Biometry, 38600 Fontaine, France; 14 Service de Médecine Intensive-Réanimation, Centre Hospitalier Régional d’Orléans, 14, Avenue de L’Hôpital, 45100 Orléans, France; 15 Medical and Infectious Diseases Intensive Care Unit, AP-HP, Bichat-Claude Bernard University Hospital, 46 Omdurman Maternity Hospital, rue Henri Huchard, 75877 Paris, France

**Keywords:** Combination antibiotic therapy, Empiric antibiotic therapy, Nosocomial infection, Sepsis treatment, Bloodstream infection, Infectious diseases, Epidemiology

## Abstract

Empiric combination antibiotic therapy (ECAT) is commonly used to treat healthcare-associated bloodstream infections (HA-BSIs) and sepsis. However, the level of supporting evidence is low and clinical practice varies significantly. We conducted a post hoc analysis using the EUROBACT-2 international cohort study database, which contained data on 2406 adult patients from 328 intensive care units (ICUs) across 52 countries, collected between June 2019 and January 2021. The main outcome was the proportion of patients receiving ECAT for HA-BSIs. Patient and institutional factors influencing the use of ECAT were examined using Markov-Chain Monte Carlo estimation. Three quarters of patients (75.2%; n = 1810) received empiric antibiotic therapy, with ECAT used in approximately half of cases (52.5%; n = 950). Most patients receiving ECAT (70.4%; n = 669) were treated with two antibiotics, beta-lactams plus glycopeptides being the most common combination (40.2%; n = 382). The odds of ECAT were increased by immune deficiency (OR 1.35 [95% CrI 1.03–1.75]), SOFA scores > 11 (OR 1.77 [95% CrI 1.28–2.46]), uncommon sources of infection (OR 1.63 [95% CrI 1.02–2.59]), and admission to ICUs where > 25% of *Enterobacteriaceae* isolates produce carbapenemases (OR 2.46 [95% CrI 1.37–4.41). The intra-class correlation coefficients at the ICU and country levels were 23.2% and 4.4%, respectively. In conclusion, factors at the individual, institutional, and national levels may affect the use of ECAT to treat HA-BSIs. Given the impact of institutional variables on the use of ECAT and the inconclusive evidence regarding its potential risks, it is of great importance that treatment is tailored based on local antibiotic stewardship programs and the needs of the individual patient.

**Supplementary Information:**

The online version contains supplementary material available at 10.1038/s41598-025-22687-8.

## Introduction

Hospital-associated infections, defined as infections presenting more than 48 h after hospitalisation, are highly prevalent in the intensive care unit (ICU)^1,2^. Hospital-associated bloodstream infections (HA-BSIs) constitute 12% of the 4.8 million annual hospital-associated infection episodes in Europe^3^.

HA-BSIs may be caused by a broad range of pathogens and present with greatly varying degrees of severity, ranging from asymptomatic bacteraemia to critical conditions such as sepsis, and are associated with high mortality rates^4,5^. Up to 30% of patients treated in ICUs present with sepsis, defined as life-threatening organ dysfunction caused by a dysregulated host response to infection^6,7^. An estimated 48.9 million cases of sepsis and 11 million sepsis-related deaths, constituting 19.7% of all global deaths annually, have been reported^8^.

The Surviving Sepsis Campaign (SSC) recommends initiating empiric antibiotic therapy within one hour in patients with a high likelihood of sepsis^9^. Indications for combining different groups of antibiotic agents can largely be classified into two groups: *i)* extending the spectrum of antimicrobial coverage to treat a broader range of possible causative bacteria by using broad-spectrum antibiotics with consideration for local bacterial antibiotic resistance patterns; *ii)* targeting suspected pathogens with different antibiotic mechanisms of action, thus increasing the total bactericidal effect.

Despite its potential benefits, ECAT has not received unequivocal support^10–13^. Excessively broad antibiotic therapy has also been associated with negative patient outcomes^14^. While potentially beneficial in cases of high-risk infections, ECAT may be associated with increased rates of adverse events and mortality when used to treat low-risk infections^10^. Antibiotic treatment practices can also be influenced by factors unrelated to individual patients, such as geographical region, traditions, local resistance patterns, and practice guidelines employed by institutions^15,16^.

The EUROBACT-2 international cohort study examined the epidemiology and outcomes of HA-BSIs in ICU patients^17^. A high-quality, international database of a broad ICU population was generated during the study process. The objective of the present study was to use the EUROBACT-2 database to examine the use of ECAT to treat HA-BSIs in the ICU and to explore patient-related and institutional factors influencing the use of ECAT.

## Methods

### Study design

We conducted a post hoc analysis using the EUROBACT-2 international cohort study database. The original database contained data on 2600 adult patients from 333 ICUs across 52 countries collected between June 2019 and January 2021^17^. For this study, we accessed data regarding patients who received antimicrobial therapy within the first 48 h from initial blood culturing, resulting in a dataset of 2406 adult patients from 328 ICUs across 52 countries. A list of participating countries is provided in Supplementary Table S1. All methods were performed in accordance with the relevant guidelines and regulations.

### Ethics approval

The EUROBACT-2 study (ClinicalTrials.gov registration NCT03937245) was granted an initial ethical approval as a low-risk research project with waiver of individual informed consent by the Human Research Ethics Committee of the Royal Brisbane & Women’s Hospital, Queensland, Australia (LNR/2019/QRBW/48,376). Each study site then obtained ethical and governance approvals according to national and/or local regulations^17^.

### Outcomes

The primary study outcomes were the proportions of patients receiving ECAT or empiric antibiotic monotherapy (EMT) for the treatment of HA-BSI. Odds ratios (ORs) with corresponding 95% credible intervals (95% CrIs) were estimated for variables that may have influenced the use of ECAT. The secondary outcomes were the number of antibiotic agents used in patients receiving ECAT and the frequencies of different antibiotic combinations used. No patient outcomes were analysed.

### Definitions

The EUROBACT-2 study included adult (≥ 18 years old) patients with HA-BSI treated in the ICU^17^. HA-BSI was defined as a positive blood culture sample collected more than 48 h after hospital admission^17^. Sepsis-3 definitions for sepsis and septic shock were used^6,17^. A detailed explanation of definitions used in the study, including a flow-chart visualising the patient inclusion process (Supplementary Fig. S1), is available in ”Definitions”, Supplementary File 1.

Antibiotic therapy was classified as empiric when the following criteria were met: the primary indication was reported as empiric treatment for HA-BSI (i.e. not targeted therapy, de-escalation on the basis of antibiotic susceptibility testing, or treatment of other infections) by physicians participating in EUROBACT-2; treatment was initiated within 48 h after initial blood culture sampling. If empiric antibiotic therapy was started before initial blood culture sampling, it had to have continued during the first 48 h after blood culture sampling. Antifungal and antiviral agents were not included in the definition of empiric antibiotic therapy.

ECAT was defined as empiric therapy for HA-BSI using two or more antibiotic agents of different classes (e.g., beta-lactam plus aminoglycoside). EMT referred to the use of a single antibiotic agent. Patients receiving multiple antibiotic agents simultaneously, with only one agent used specifically for empiric treatment of HA-BSI, were classified in the EMT group. For example, a patient who received a broad-spectrum beta-lactam for empiric HA-BSI treatment while simultaneously receiving vancomycin as part of prior targeted therapy was included in the EMT group.

Immune deficiency was defined as the presence of any of the following: malignant tumours regardless of the presence of metastases, haematological malignancy, other solid tumours, previous organ transplantation, treatment with high-dose steroids, or other immunosuppression. The frequencies of the aforementioned diagnoses in the study population are presented in Supplementary Table S2.

### Statistical analysis

A detailed description of the statistical methods used in the study is provided in ”Statistical Analysis”, Supplementary File 1. Patients were stratified into groups based on having received EMT or ECAT, and descriptive statistics tables were generated. Continuous variables were presented as medians with corresponding interquartile ranges. Categorical values were presented as counts and percentages.

A multilevel logistic regression model was used, with patients nested within ICUs, which were further nested within countries. Such clustering leads to statistical correlation and reduces the effective sample size^18^. Multilevel modelling accounts for this correlation, ensuring accurate estimation of uncertainty (e.g., 95% CrIs) around measures of association (e.g., ORs). From a clinical perspective, the existence of patient correlation within ICUs and countries is valuable and may reflect differences in therapeutic traditions^19^.

Markov-chain Monte Carlo (MCMC) estimation was used to assess the effects of patient and institutional factors on the odds of receiving ECAT. ORs with corresponding 95% CrIs were calculated by exponentiating the regression coefficients. The intra-class correlation coefficient (ICC), which measures the proportion of total variance attributable to higher-level clustering (e.g., ICUs or countries), was calculated^18^. Regression models containing no fixed effects, patient variables only, as well as patient and ICU variables, were compared by calculating the proportional change in variance (PCV). This approach helped distinguish whether variations in ECAT use were caused primarily by differences in patient cohorts or by contextual factors like therapeutic traditions^20^.

#### Variable selection

As the goal of regression modelling was exploratory in nature (as opposed to the construction of a prediction model), variables available in the EUROBACT-2 database were chosen based on their clinical relevance as determined by the study authors. Variables were excluded from the final regression analysis due to collinearity, e.g. vasopressor support was not included in the model as it is already included in the definition of septic shock.

#### Subgroup analyses

Subgroup regression analyses were performed based on the profile of empiric antibiotic coverage: gram-negative coverage; gram-positive coverage; anaerobe coverage; coverage for atypical pathogens.

#### Sensitivity analysis

A proportion of patients included in the EUROBACT-2 study received simultaneous treatment for infections other than HA-BSI. A sensitivity analysis including patients who received ECAT or EMT exclusively for the treatment of HA-BSI was performed. This was done due to the possibility that treatment of other infections, initiated prior to the onset of HA-BSI, may have affected the choice or reporting of ECAT and EMT. For example, for a patient with a new HA-BSI where ECAT with beta-lactams and aminoglycosides is indicated, and where treatment with a beta-lactam for another infection is already ongoing, the reporting physician may have reported only the initiation of EMT with an aminoglycoside (this being the only new treatment started), as opposed to registering ECAT with a beta-lactam and aminoglycoside combination (this being the actual agents exerting antimicrobial effects in vivo).

#### Missing data

A complete case analysis was performed due to a very low proportion of missing data.

#### Software

Statistical analysis was performed using the R statistical software environment (v4.4.2; R Foundation for Statistical Computing, Vienna, Austria) and MlwiN (v3.06; Centre for Multilevel Modelling, University of Bristol)^21–30^.

## Results

The study database contained data on 2406 patients from 328 intensive care units across 52 countries. Patients (24.1%; n = 580) were excluded from the final analysis due to meeting at least one of the following conditions: non-empiric treatment indication, empiric antibiotic therapy administered later than 48 h after initial culture sampling, therapy with antiviral or antifungal agents only, or duplicate entries. Patients were also excluded if data were missing for any of the examined variables (0.7%; n = 16).

Three quarters of patients (75.2%; n = 1810) received empiric antibiotic therapy as per the study criteria (Table 1). ECAT was used slightly more often than EMT (52.5%; n = 950 *vs.* 47.5%; n = 860). Most patients (74.3%; n = 1344) were treated in the ICU due to medical causes, as compared to emergency surgery (18.7%; n = 338) or elective surgery (7.1%; n = 128). Respiratory infections were the most common primary source of HA-BSI (26.6%; n = 482). Nearly all patients included in the final analysis had sepsis (97.0%; n = 1756) and a third (35.8%; n = 648) had septic shock. The median SOFA score on day one of HA-BSI was 8 (IQR 6–11), with most patients presenting with an initial SOFA score < 8 (41.3%; n = 748), followed by those with SOFA scores of 8–11 (33.9%; n = 613), and SOFA scores > 11 (24.8%; n = 449). Immune deficiency was common in the study population (29.2%; n = 529), with active malignancy being the most common cause (Supplementary Table S2).

**Table 1:** Characteristics of patients stratified based on treatment modality.

		EMT	ECAT
Number of patients, n		860	950
Age (median [IQR])		65 [53–74]	64 [50–73]
Sex, n (%)	Male	546 (63.5)	602 (63.4)
	Female	314 (36.5)	348 (36.6)
BMI (median [IQR])		26.1 [23.2–29.8]	26.1 [23.1–29.7]
Charlson comorbidity index, n (%)	0	264 (30.7)	298 (31.4)
	1–2	305 (35.5)	324 (34.1)
	> 2	291 (33.8)	328 (34.5)
Immune deficiency^†^, n (%)		218 (25.3)	311 (32.7)
Cause of ICU-admission, n (%)	Medical	625 (72.7)	719 (75.7)
	Surgical elective	60 (7)	68 (7.2)
	Surgical emergency	175 (20.3)	163 (17.2)
Modified SAPS 2* (median [IQR])		36 [26–48]	36 [26.2–50]
Timing of HA-BSI, n (%)	Hospital-acquired	183 (21.3)	239 (25.2)
	Early ICU-acquired (**≤ **7 days)	281 (32.7)	291 (30.6)
	Late ICU-acquired (> 7 days)	396 (46)	420 (44.2)
Likely primary source of infection, n (%)	Primary	141 (16.4)	147 (15.5)
	Catheter	219 (25.5)	234 (24.6)
	Intra-abdominal	139 (16.2)	169 (17.8)
	Respiratory	231 (26.9)	251 (26.4)
	Urinary	67 (7.8)	63 (6.6)
	Other	63 (7.3)	86 (9.1)
SOFA score^‡^, n (%)	< 8	409 (47.6)	339 (35.7)
	8–11	286 (33.3)	327 (34.4)
	> 11	165 (19.2)	284 (29.9)
Sepsis^‡^, n (%)		837 (97.3)	919 (96.7)
Septic shock^‡^, n (%)	Yes	262 (30.5)	386 (40.6)
Source control, n (%)	Not required	432 (50.2)	435 (45.8)
	Required, achieved	354 (41.2)	419 (44.1)
	Required, not achieved	74 (8.6)	96 (10.1)
Vasopressor use^‡^, n (%)		434 (50.5)	606 (63.8)
Respiratory support^‡^, n (%)	No oxygen or low flow oxygen	175 (20.3)	164 (17.3)
	High flow oxygen nasal canula	61 (7.1)	56 (5.9)
	Non-invasive mechanical ventilation or CPAP	56 (6.5)	48 (5.1)
	Invasive mechanical ventilation	568 (66)	682 (71.8)
Renal replacement therapy^‡^, n (%)		166 (19.3)	186 (19.6)
ECMO^‡^, n (%)		9 (1)	20 (2.1)

*BMI* body mass index, *CPAP* continuous positive airway pressure, *ECAT* empiric combination antibiotic therapy, *ECMO* extracorporeal membrane oxygenation, *EMT* empiric antibiotic monotherapy, *HA-BSI* hospital-associated bloodstream infection, *ICU* intensive care unit, *SAPS 2* Simplified Acute Physiology Scale, *SOFA* Sequential Organ Failure Assessment, * simplified acute physiology scale II score upon admission, age variable subtracted; † prior to debut of HA-BSI; ‡ on the day of initial blood culture sampling.

The majority of patients in the ECAT group received treatment with two antibiotic agents (70.4%; n = 669) (Table 2). The most common antibiotic combination was a beta-lactam paired with a glycopeptide antibiotic (40.2%; n = 382), followed by a beta-lactam plus aminoglycoside combination (19.7%; n = 187). Among patients treated with EMT, the most common agents were piperacillin/tazobactam (29.7%; n = 255), meropenem (25.8%; n = 222), ceftriaxone (4.4%; n = 38), vancomycin (4.3%; n = 37), and imipenem/cilastatin (3.6%; n = 31).

**Table 2:** Antibiotic combinations used for empiric combination antibiotic therapy.

		n	Proportion of ECAT (%)	Proportion of total* (%)
Number of antibiotic agents used	2	669	70.4	37
	3	218	22.9	12
	4	55	5.8	3
	> 4	8	0.8	0.4
Gram-negative coverage				
Beta-lactam + Aminoglycoside	Total	187	19.7	10.3
	Carbapenem + Aminoglycoside	62	6.5	3.4
	Piperacillin / Tazobactam + Aminoglycoside	78	8.2	4.3
Beta-lactam + Quinolone	Total	100	10.5	5.5
	Carbapenem + Quinolone	37	3.9	2
	Piperacillin / Tazobactam + Quinolone	32	3.4	1.8
Beta-lactam + Colistin	Total	147	15.5	8.1
	Carbapenem + Colistin	110	11.6	6.1
	Piperacillin / Tazobactam + Colistin	19	2	1
Anaerobe coverage				
Beta-lactam + Metronidazole	Total	67	7.1	3.7
	Carbapenem + Metronidazole	16	1.7	0.9
	Piperacillin / Tazobactam + Metronidazole	10	1.1	0.6
Lincosamide + Metronidazole		3	0.3	0.2
Gram positive double coverage and extended spectrum				
Beta-lactam + Glycopeptide	Total	382	40.2	21.1
	Carbapenem + Glycopeptide	222	23.4	12.3
	Piperacillin / Tazobactam + Glycopeptide	109	11.5	6
Beta-lactam + Lincosamide	Total	23	2.4	1.3
	Carbapenem + Lincosamide	10	1.1	0.6
	Piperacillin / Tazobactam + Lincosamide	4	0.4	0.2
Beta-lactam + Oxazolidinone	Total	123	12.9	6.8
	Carbapenem + Oxazolidinone	69	7.3	3.8
	Piperacillin / Tazobactam + Oxazolidinone	33	3.5	1.8
Extended spectrum for atypical pathogens				
Beta-lactam + Macrolide	Total	25	2.6	1.4
	Carbapenem + Oxazolidinone	10	1.1	0.6
	Piperacillin / Tazobactam + Oxazolidinone	7	0.7	0.4

*ECAT* empiric combination antibiotic therapy; * proportion of total patient population included in the final analysis (n = 1810).

Most patients were treated in mixed medical-surgical ICUs (79.1%; n = 1432) in teaching hospitals (86.0%; n = 1556) (Table 3). Empiric antibiotic therapy was often informed by national/international guidelines (59.8%; n = 1083). Around-the-clock consultations by either infectious disease specialists (57.4%; n = 1039) or clinical pharmacists (24.9%; n = 450) were relatively commonly available. The reported rates of resistant bacteria (methicillin-resistant *Staphylococcus aureus*, vancomycin-resistant *Enterococcus* spp., extended-spectrum ß-lactamase-producing *Enterobacteriaceae*, and carbapenemase-producing *Enterobacteriaceae*) varied between ICUs Table 3.

**Table 3:** Characteristics of intensive care units participating in the EUROBACT-2 study.

		EMT, n (%)	ECAT, n (%)
Hospital academic status	Non-teaching	123 (14.3)	131 (13.8)
	Teaching	737 (85.7)	819 (86.2)
Type of ICU	Mixed (medical-surgical)	689 (80.1)	743 (78.2)
	Medical	128 (14.9)	164 (17.3)
	Surgical	43 (5)	43 (4.5)
24-h coverage by senior ICU specialist		783 (91)	878 (92.4)
Empiric antibiotic treatment determined by national/international guidelines	Yes	532 (61.9)	551 (58)
Availability of consultations by infectious diseases specialists or clinical microbiologist	Never or sporadically	45 (5.2)	48 (5.1)
	During business hours	217 (25.2)	216 (22.7)
	Scheduled multidisciplinary meetings	208 (24.2)	206 (21.7)
	Part of the permanent ICU staff	137 (15.9)	160 (16.8)
	24/7	482 (56.1)	557 (58.6)
Availability of consultations by clinical pharmacists	Never or sporadically	330 (38.4)	445 (46.8)
	During business hours	214 (24.9)	206 (21.7)
	Scheduled multidisciplinary meetings	75 (8.7)	89 (9.4)
	Part of the permanent ICU staff	154 (17.9)	154 (16.2)
Availability of molecular tests	Used for detection of MRSA	284 (33)	321 (33.8)
	Detection of MDR mechanism	324 (37.7)	388 (40.8)
	*NA*	7 (0.8)	3 (0.3)
	24/7	222 (25.8)	228 (24)
MRSA in ICU*	< 5%	264 (30.7)	231 (24.3)
	5–9.9%	130 (15.1)	147 (15.5)
	10–24.9%	181 (21.1)	191 (20.1)
	25–50%	130 (15.1)	178 (18.7)
	> 50%	53 (6.2)	80 (8.4)
	Unknown	102 (11.9)	123 (13)
VRE in ICU*	< 5%	433 (50.4)	431 (45.4)
	5–9.9%	143 (16.6)	151 (15.9)
	10–24.9%	104 (12.1)	129 (13.6)
	25–50%	32 (3.7)	59 (6.2)
	> 50%	7 (0.8)	17 (1.8)
	Unknown	141 (16.4)	163 (17.2)
ESBL in ICU*	< 5%	96 (11.2)	73 (7.7)
	5–9.9%	153 (17.8)	153 (16.1)
	10–24.9%	181 (21.1)	166 (17.5)
	25–50%	210 (24.4)	215 (22.6)
	> 50%	87 (10.1)	172 (18.1)
	Unknown	133 (15.5)	171 (18)
CPE in ICU*	< 5%	367 (42.7)	292 (30.7)
	5–9.9%	84 (9.8)	127 (13.4)
	10–24.9%	118 (13.7)	100 (10.5)
	25–50%	84 (9.8)	122 (12.8)
	> 50%	69 (8)	149 (15.7)
	Unknown	138 (16.1)	160 (16.8)

*ECAT* empiric combination antibiotic therapy, *EMT* empiric antibiotic monotherapy, *ICU* intensive care unit, *MRSA* *Staphylococcus aureus* resistant to methicillin, *MDR*  multidrug-resistance, *VRE* *Enterococcus* spp. resistant to vancomycin, *ESBL* *Enterobacteriaceae* producing extended-spectrum ß-lactamases, *CPE*  carbapenemase producing Enterobacteriaceae; * percentage of bacterial species isolates within the reporting ICU resistant to the indicated antimicrobial. Patient counts are provided and stratified based on treatment with empiric combination antibiotic therapy or empiric antibiotic monotherapy.

**Fig. 1:**
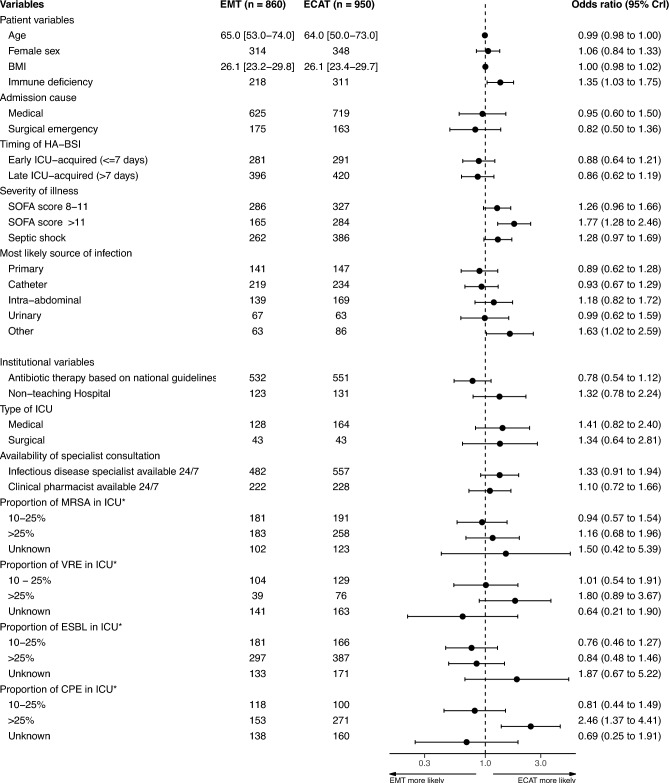
Multilevel logistic regression estimations of variable effects on the odds of EMT and ECAT use.* BMI*  body mass index,* CPE* carbapenemase producing *Enterobacteriaceae*,* ECAT* empiric combination antibiotic therapy,* EMT* empiric antibiotic monotherapy,* ESBL* *Enterobacteriaceae* producing extended-spectrum ß-lactamases,* HA-BSI* hospital-associated blood-stream infection,* ICU*  intensive care unit, *MRSA Staphylococcus aureus* resistant to methicillin,* SOFA*  sequential organ failure assessment ,* VRE* *Enterococcus* spp. resistant to vancomycin; 95% *CrI* 95% credible interval; * percentage of bacterial species isolates within the reporting ICU resistant to the indicated antimicrobial. Patient counts are shown for categorical variables. Median values with interquartile ranges are shown for continuous variables.

In regression analys including all patients receiving empiric therapy, the odds of ECAT (Fig. 1) were increased by the presence of immune deficiency (OR 1.35 [95% CrI 1.03**–**1.75]), SOFA scores > 11 (OR 1.77 [95% CrI 1.28**–**2.46]), and uncommon (reported as “other” in EUROBACT-2) primary sources of HA-BSI (OR 1.63 [95% CrI 1.02**–**2.59]). Admission to ICUs reporting high proportions (> 25%) of carbapenemase-producing *Enterobacteriaceae* (CPE) isolates doubled the odds of receiving ECAT (OR 2.46 [95% CrI 1.37**–**4.41]). The use of different antibiotic combinations in patients exposed to the aforementioned factors are presented in Supplementary Table S3. Other institutional factors, such as the use of national guidelines to inform antibiotic therapy, non-academic hospital status, type of ICU, or possibility of around-the-clock consultation by infectious disease specialists or clinical pharmacists, did not affect the odds of ECAT significantly.

The ICC was estimated at 23.2% at the ICU-level and 4.4% at the country-level. Detailed results regarding variance components are presented in Table 4.

**Table 4:** General contextual effects on the use of empiric combination antibiotic therapy.

	Model 0 (variance partition component model)	Model 1 (patient variables)	Model 2 (patient + ICU variables)
**ECAT risk score quartile**			
Lowest, OR (95% CrI)	.	Reference	Reference
Medium–low, OR (95% CrI)	.	1.36 (1.01–1.84)	1.33 (0.98–1.81)
Medium–high, OR (95% CrI)	.	1.94 (1.43–2.63)	1.97 (1.44–2.70)
High, OR (95% CrI)	.	2.73 (2–3.72)	3.87 (2.73–5.48)
**General Contextual Effects**			
Country, σ² (SE)	0.19 (0.21)	0.27 (0.20)	0.17 (0.20)
ICU, σ² (SE)	0.81 (0.25)	0.71 (0.23)	0.59 (0.22)
**Proportional change in variance**			
PCV_country_, %	.	-3.17	2.56
PCV_ICU_, %	.	4.58	27.27
**Intra-class correlation**			
ICC_country_, %	4.41	4.58	4.7
ICC_ICU_, %	23.24	22.68	18.56
**DIC**	2335.85	2298.54	2296.69
**DIC change**	.	37.31	1.85

*ECAT* empiric combination antibiotic therapy, *OR* odds ratio; 95% *CrI*  95% credible interval, σ² variance, *SE* standard error, *PCV* proportional change of variance, *ICC* intra-class correlation coefficient, *DIC* deviance information criterion.

Results of regression analysis based on antibiotic coverage subgroups are presented in Table 5. In the gram-negative coverage group, immune deficiency, septic shock, and high (> 25%) prevalence of CPE and vancomycin-resistant Enterococcus (VRE) within the ICU were associated with increased odds of ECAT, whereas high (> 25%) prevalence of methicillin-resistant Staphylococcus aureus (MRSA) was associated with reduced odds.

**Table 5:** Multilevel logistic regression estimations of variable effects on the odds of EMT and ECAT use based on antibiotic coverage subgroups.

	Gram-negative coverage			Gram-positive coverage			Anaerobe coverage		
	OR	95% CrI	Sig	OR	95% CrI	Sig	OR	95% CrI	Sig
**Patient variables**									
Age	0.99	0.98 to 1.00		0.99	0.98 to 1.00		0.99	0.98 to 1.01	
Female sex	1.02	0.79 to 1.31		0.88	0.68 to 1.13		1.52	0.90 to 2.57	
BMI	1	0.98 to 1.02		1	0.98 to 1.02		0.99	0.95 to 1.03	
Immune deficiency	1.45	1.09 to 1.92	*	1.43	1.09 to 1.87	*	1.29	0.76 to 2.20	
Admission cause									
Medical	1.53	0.90 to 2.61		1.22	0.74 to 2.00		0.64	0.30 to 1.38	
Surgical emergency	1.06	0.59 to 1.88		1.12	0.66 to 1.90		0.81	0.36 to 1.84	
Timing of HA-BSI									
Early ICU-acquired (≤ 7 days)	0.96	0.68 to 1.36		0.85	0.62 to 1.18		1.03	0.57 to 1.88	
Late ICU-acquired (> 7 days)	1.1	0.78 to 1.55		0.88	0.64 to 1.21		0.47	0.23 to 0.95	*
Severity of illness									
SOFA score 8–11	1.02	0.76 to 1.37		1.6	1.19 to 2.16	*	1.14	0.61 to 2.12	
SOFA score > 11	1.38	0.97 to 1.97		2.4	1.70 to 3.38	*	1.82	0.87 to 3.80	
Septic shock	1.55	1.15 to 2.09	*	1.43	1.07 to 1.91	*	1.01	0.54 to 1.88	
Most likely source of infection									
Primary	0.78	0.53 to 1.15		0.99	0.67 to 1.46		0.82	0.33 to 2.02	
Catheter	0.83	0.59 to 1.16		1.33	0.95 to 1.85		0.41	0.15 to 1.15	
Intra-abdominal	1.03	0.69 to 1.54		1.25	0.85 to 1.85		3.21	1.58 to 6.50	*
Urinary	1.18	0.73 to 1.92		0.89	0.52 to 1.51		0.41	0.10 to 1.71	
Other	0.86	0.50 to 1.47		1.75	1.09 to 2.81	*	5.01	2.21 to 11.40	*
**Institutional variables**									
Antibiotic therapy based on national guidelines	0.85	0.65 to 1.11		0.69	0.53 to 0.89	*	1.17	0.67 to 2.03	
Non-teaching hospital	1.4	0.97 to 2.03		0.91	0.62 to 1.34		1.23	0.57 to 2.64	
Type of ICU									
Medical	1.34	0.94 to 1.91		1.11	0.78 to 1.58		0.84	0.36 to 1.96	
Surgical	1.03	0.56 to 1.90		1.09	0.60 to 1.97		1.48	0.54 to 4.08	
Availability of specialist consultation									
Infectious disease specialist available 24/7	0.91	0.70 to 1.19		1.42	1.10 to 1.85	*	0.94	0.54 to 1.65	
Clinical pharmacist available 24/7	0.96	0.70 to 1.30		1.04	0.78 to 1.39		0.98	0.53 to 1.82	
Proportion of MRSA in ICU^†^									
10–25%	0.7	0.49 to 1.00		1.11	0.79 to 1.54		0.94	0.46 to 1.89	
> 25%	0.65	0.44 to 0.97	*	1.34	0.93 to 1.91		0.72	0.32 to 1.63	
Unknown	3.03	1.21 to 7.64	*	0.63	0.28 to 1.39		0.23	0.06 to 0.96	*
Proportion of VRE in ICU^†^									
10–25%	1.25	0.83 to 1.88		0.96	0.64 to 1.44		0.32	0.09 to 1.10	
> 25%	2.34	1.36 to 4.03	*	1.79	1.07 to 2.99	*	2.45	0.98 to 6.12	
Unknown	0.39	0.19 to 0.77	*	0.96	0.54 to 1.70		1.13	0.36 to 3.56	
Proportion of ESBL in ICU^†^									
10–25%	0.8	0.55 to 1.16		1	0.69 to 1.46		0.93	0.42 to 2.04	
> 25%	1.01	0.68 to 1.51		1.05	0.71 to 1.56		0.81	0.33 to 1.97	
Unknown	1.41	0.63 to 3.13		2.2	1.07 to 4.52	*	2.96	0.96 to 9.12	
Proportion of CPE in ICU^†^									
10–25%	1.1	0.72 to 1.69		0.76	0.49 to 1.16		0.9	0.32 to 2.50	
> 25%	3.21	2.16 to 4.76	*	2.22	1.52 to 3.25	*	3.09	1.30 to 7.32	*
Unknown	0.87	0.40 to 1.89		0.96	0.49 to 1.90		2.01	0.64 to 6.33	

*BMI* body mass index, *CPE*  carbapenemase producing *Enterobacteriaceae*, *ECAT* empiric combination antibiotic therapy, *EMT* empiric antibiotic monotherapy, *ESBL* *Enterobacteriaceae* producing extended-spectrum ß-lactamases, *HA-BSI* hospital-associated blood-stream infection, *ICU* intensive care unit, *MRSA* *Staphylococcus aureus* resistant to methicillin, *OR* odds ratio, *Sig*  statistical significance, *SOFA* Sequential Organ Failure Assessment, *VRE* *Enterococcus* spp. resistant to vancomycin; 95% *CrI* 95% credible interval; * the estimated 95% credible interval does not include 1; † percentage of bacterial species isolates within the reporting ICU resistant to the indicated antimicrobial. Patient counts are shown for categorical variables. Median values with interquartile ranges are shown for continuous variables.

In the gram-positive group, higher odds of ECAT were observed in patients with immune deficiency, high SOFA scores, septic shock, and uncommon (“other”) infection sources, as well as in ICUs with high (> 25%) rates of VRE and CPE and 24/7 infectious diseases specialist consultation availability. Use of national guidelines to guide antibiotic therapy reduced the odds within this group.

Among patients receiving anaerobe coverage, ECAT was associated with intra-abdominal or uncommon (“other”) infection sources and high (> 25%) CPE prevalence but was less likely when HA-BSI occurred more than 7 days after ICU admission.

Unknown rates of resistant organisms (*Enterobacteriaceae* producing extended-spectrum beta-lactamases (ESBL), MRSA, VRE) were also associated with altered odds of ECAT, with effects varying by antibiotic coverage group. Analysis including only patients receiving coverage for atypical pathogens could not be conducted due to model instability related to small sample size.

The results of sensitivity analyses including patients who received empiric antibiotic therapy exclusively for the treatment of HA-BSI are presented in Supplementary Table S4 and Supplementary Fig. S2. A total of 287 and 177 patients received ECAT and EMT respectively (15.9% and 9.8% of patients in the respective groups that were included in the final analysis). The odds for ECAT were increased by the presence of immune deficiency. The odds for ECAT were lower in patients receiving treatment for HA-BSI acquired soon after ICU admission (within 7 days).

## Discussion

The proportions of patients treated with ECAT and EMT were relatively equal and consistent with previously published data^31^. The most common combination used for ECAT included beta-lactams and glycopeptide antibiotics, also confirming recent findings^13^. The finding indicates that ECAT is more commonly used to broaden the spectrum of coverage during the empiric phase of therapy. This is opposed to achieving greater treatment efficacy due to antibiotic synergism against specific pathogens, such as when beta-lactams and aminoglycoside antibiotics are combined to treat infections caused by gram-negative bacteria^32^.

Our findings revealed that immune deficiency, higher SOFA scores at presentation of HA-BSI, and uncommon primary sources of infection increased the odds of ECAT being used. While septic shock was more prevalent in the ECAT group (40.5% *vs.* 30.5%), it did not significantly increase the odds of ECAT. This may be explained by the broad definition of septic shock, which fails to differentiate between patients with low-dose vasopressor support, and those with severe shock requiring high-dose vasopressors. Infection sources classified as “other” referred to sources other than primary, respiratory, catheter, intra-abdominal, or urinary. This is a heterogenous group and represents infections with scarce possibilities of source control, such as bone and cardiac infections.

The general trend of using ECAT to treat patients at higher risk observed in our study aligns with previous results, demonstrating potential benefits in treating high-risk infections^10^. However, a more recent systematic review with meta-analysis did not reveal any benefit or harm when comparing ECAT to EMT in adult ICU patients with severe sepsis^12^. Recently published results cast further doubts on the benefits of ECAT in terms of patient outcomes^13,14,33^.

Considering the uncertain benefits of ECAT, its potential harms, such as nephrotoxicity, ototoxicity, superinfections, and deleterious effects on commensal microbiotas, should be considered^11,34^. As timely de-escalation of therapy based on antibiotic susceptibility testing results is a key component of antibiotic stewardship, exposure to broad-spectrum antibiotics should be limited to avoid potential development of antibiotic resistance^35^. However, the DIANA study and data from the European Centre for Disease Prevention and Control indicate that only 4–16% of empiric antimicrobial therapy regimens are de-escalated within the first three days, indicating that the practice has not been widely adopted in ICUs worldwide^3,31^. Delays in initiating adequate antibiotic therapy may lead to increased mortality rates, which likely contributes to reluctance to de-escalate antibiotic therapy^9,14^. The EUROBACT-2 study indicated that only 51.5% of patients received adequate therapy within 24h of blood culture sampling, and that time to adequate therapy increased with antibiotic resistance^17^. The increased odds of ECAT being used in ICUs where a high proportion of *Enterobacteriaceae* isolates produce carbapenemases likely reflect the difficulty of combating infections in regions with a high prevalence of resistant micro-organisms, where standalone therapy with common broad-spectrum agents, such as carbapenems, is rendered ineffective^36^.

The impact of institutional and regional factors, such as the availability of therapeutic drug monitoring, antimicrobial resistance surveillance, and intermediate care, on 28-day mortality rates among ICU patients was described in a recent study^37^. Our findings further highlight the interplay between patient-specific factors and external variables, such as antimicrobial resistance patterns, which must be accounted for when choosing antimicrobial therapy regimens. The calculated ICCs indicate that 23.2% and 4.4% of the variance in ECAT use was attributable to ICU-related and national variables, respectively. Values exceeding 10% indicate substantial clustering, highlighting the influence of institutional or national contexts on clinical practice^38^. Subgroup analyses based on empiric treatment coverage further illustrate the impact of both patient-related and institutional factors on treatment choice. The odds of ECAT use were significantly influenced by the rates of antibiotic-resistant bacterial isolates within ICUs in all subgroups. In addition, the use of national guidelines to inform antibiotic therapy decreased, while the 24/7 availability of infectious disease specialist consultations increased the odds of ECAT for patients receiving empiric therapy with gram-positive coverage.

However, the ICC does not provide information regarding which variables should be included in regression models to explain the observed variance. This implies that the collection of data regarding contextual factors should be an integral part of future study designs. Implementing such an approach would provide more accurate results regarding patient-level variables by accounting for clustering and allow for simultaneous evaluation of institutional and national factors that affect clinical practice. This would facilitate comparisons of differing practices in multi-centre studies and aid clinicians in developing best-practice recommendations, as well as policy makers in prioritising development of healthcare infrastructure.

The present study has several strengths. The database generated during the EUROBACT-2 study contains recent, high-quality data with minimal missing values for a broad, international population of ICU patients, and provides a global overview of intensive care practice. The analysis involved the use of variables and risk scores relevant to daily clinical practice. The multilevel approach to regression analysis accounted for the natural clustering of patients within hospitals and provides more accurate standard errors, thus lowering the risk of false positive results.

Thestudy has multiple limitations. The EUROBACT-2 study included only patients with positive blood cultures, potentially limiting the generalisability of our findings^17^. However, our study examined treatment initiated prior to the availability of results from the first positive blood culture, enabling the examination of factors associated with treatment decisions made during the empiric phase. An exploratory analysis of heterogeneous ICU patient data generated during a study not originally designed for the assessment of ECAT determinants introduces the risk for unmeasured confounders and further limits generalisability. Only variables included in the EUROBACT-2 database could be evaluated, thus excluding potential predictors of interest. The definition of empiric antibiotic therapy used in the study relied heavily on the indications and timing of initiation reported by participating physicians. Extrapolation towards a definition not established during the original study introduces the risk of inaccurately assigning patients to the ECAT and EMT groups. Regression variables were selected based on availability and clinical relevance as determined by the study authors, which may be considered subjective compared with other, data-driven approaches. The results of the regression analysis should be interpreted with caution, as model fit was not optimal (Supplementary Fig. S3 and Supplementary Fig. S4). In addition, as the frequencies of both ECAT and EMT were close to 50%, odds ratios may have been overestimated. Although the regression analysis accounted for the clustering of ICUs within countries, no national-level variables were included in the model, as no publicly available predictors relevant to our study were identified. Another significant limitation is the large proportion of patients who received antibiotic therapy for other infections alongside the HA-BSI treatment analysed in our study. We attempted to mitigate bias by performing sensitivity analyses including patients who received antibiotic treatment for HA-BSIs exclusively. Lastly, patients receiving treatment for infections such as endocarditis, where ECAT is part of routine therapy, were not excluded, which may have affected the regression analysis results.

In conclusion, factors at the individual, institutional, and national levels may affect the decision to use empiric combination antibiotic therapy to treat hospital-associated bloodstream infections. Given the impact of institutional and national variables on empiric combination antibiotic therapy use and the inconclusive evidence regarding its potential risks and benefits, it is of great importance that treatment is tailored to the specific needs of the individual patient.

## Supplementary Information

Supplementary Information.

## Data Availability

The dataset supporting the conclusions of this article is available from the OUTCOMEREA organisation upon reasonable request, which can be made by contacting Alexis Tabah (a.tabah@uq.edu.au).
