# Supplementary material for: Determinants of empiric combination antibiotic therapy for hospital associated bloodstream infections in the intensive care unit
Source: Sci Rep. 2025 Oct 20;15:36481. doi: 10.1038/s41598-025-22687-8 (PMC12537954; doi:10.1038/s41598-025-22687-8)
Supplement: Supplementary file 1 — Supplementary Information. [file 41598_2025_22687_MOESM1_ESM.pdf]

Supplementary materials for

“Determinants of empiric combination antibiotic therapy for hospital-associated bloodstream infections in the intensive care unit”

Evaldas Kauzonas (1, 2); Gustav Torisson (3, 4); Juan Merlo (5, 6); Raquel Perez (5, 7); Alexis Tabah (8, 9, 10); Niccolò Buetti (11, 12); Stéphane Ruckly (12, 13); François Barbier (14); Jean-François Timsit, (12, 15); Fredrik Sjövall (1, 2); on behalf of the EUROBACT-2 Study Group.

Affiliations

1. Department of Clinical Sciences, Faculty of Medicine, Lund University, Sölvegatan 19, 221 84, Lund, Sweden.
2. Department of Intensive and Perioperative Care, Skåne University Hospital Malmö, Inga Marie Nilssons gata 47, 214 28, Malmö, Sweden.
3. Clinical Infection Medicine, Department of Translational Medicine Malmö, Lund University, Ruth Lundskogs gata 3, 214 28, Malmö.
4. Department of Infectious Diseases, Skåne University Hospital Malmö, Inga Marie Nilssons gata 47, 214 28, Malmö, Sweden.
5. Unit for Social Epidemiology, Faculty of Medicine, Lund University, Jan Waldenströms gata 35, 205 02, Malmö, Sweden.
6. Centre for Primary Health Care Research, Region Skåne, Jan Waldenströms gata 35, 205 02 Malmö, Sweden.
7. Department of Translational Medicine-Hand Surgery, Lund University, Skåne University Hospital, Jan Waldenströms Gata 5, 205 02, Malmö, Sweden.
8. Intensive Care Unit, Redcliffe Hospital, Metro North Hospital and Health services, Queensland, Australia.
9. Queensland University of Technology, Brisbane, Queensland.
10. Faculty of Medicine, The University of Queensland, Brisbane, Queensland.
11. Infection Control Program and WHO Collaborating Centre, Geneva University Hospitals and Faculty of Medicine, Rue Gabrielle-Perret-Gentil 4, 1205 Geneva, Switzerland.
12. Université de Paris, INSERM, IAME UMR 1137, F-75018 Paris, France.
13. ICUREsearch, Biometry, 38600 Fontaine, France.
14. Service de Médecine Intensive-Réanimation, Centre Hospitalier Régional d'Orléans, 14, avenue de l'Hôpital, 45100, Orléans, France.
15. Medical and Infectious Diseases Intensive Care Unit, AP-HP, Bichat-Claude Bernard University Hospital, 46 Omdurman maternity hospital, rue Henri Huchard, 75877, Paris Cedex, France.

Table of contents

The EUROBACT-2 Study Group....3

Participating Countries....20

Definitions....22

Statistical Analysis....24

Empiric Combination Antibiotic Therapy Stratified By Exposure....30

Sensitivity Analysis Results....31

Testing Model Fit....33

References....34

The EUROBACT-2 study group: national coordinators, scientific committee, and participating intensive care units.

East Asia and Pacific

Australia

National Coordinator: A/Prof. Alexis Tabah

Scientific Committee: Prof. Jeffrey Lipman

Participating ICUs: Redcliffe Hospital, ICU: A/Prof. Alexis Tabah, Dr Hamish Pollock, Dr Ben Margetts. Alfred Hospital, Department of Intensive Care and Hyperbaric Medicine: Prof Andrew Udy, Ms Meredith Young. Ipswich Hospital, Intensive Care Unit: Dr Neeraj Bhadange, Mr Steven Tyler. Mater Hospital, And Mater Research Institute – The University of Queensland, Mater Misericordiae Limited, The Department of Intensive Care: Dr Anne Ledtischke, Miss Mackenzie Finnis. Mater Private Hospital, And Mater Research Institute – The University of Queensland, Mater Misericordiae, The Department of Intensive Care: Dr Anne Ledtischke, Miss Mackenzie Finnis. Bankstown-Lidcombe Hospital, Intensive Care Unit: Dr Jyotsna Dwivedi, Dr Manoj Saxena. Lyell Mcewin Hospital, Lyell Mcewin Hospital Intensive Care Unit: Dr Vishwanath Biradar, Mrs Natalie Soar. Cabrini Hospital, Intensive Care: A/Prof Vineet Sarode, A/Prof David Brewster. St John Of God Murdoch Hospital, Intensive Care Unit: A/Prof Adrian Regli, Dr Elizabeth Weeda. Royal Brisbane and Women S Hospital, Intensive Care Services: Dr Samiul Ahmed, Ms Cheryl Fourie, Prof. Kevin Laupland. The Prince Charles Hospital, Adult Intensive Care Services: Dr Mahesh Ramanan. Princess Alexandra Hospital, Intensive Care: Dr James Walsham, Mr Jason Meyer. Fiona Stanley Hospital, Intensive Care Unit: Dr Edward Litton, Ms Anna Maria Palermo, Mr Timothy Yap, Mr Ege Eroglu. Rockhampton Hospital, Intensive Care Unit: Dr Antony George Attokaran, Dr C'havala Jaramillo.

Brunei

National Coordinator: Dr. Khalid Mk Nafees

Participating ICUs: Ripas Hospital, Icu 3: Dr Khalid Mahmood Khan Nafees. Raja Isteri Pengiran Anak Saleha Hospital, Icu1: Dr Nurhikmahtul Aqilah Haji Abd Rashid, Dr Haji Adi Muhamad Ibnu Walid. Gleneagles Jpmc, Icu: Dr Tomas Mon, Dr P. Dhakshina Moorthi. Suri Seri Begawan Hospital, Intensive Care Unit: Dr Shah Sudhirchandra, Dr Dhadappa Damodar Sridharan.

China

National Coordinator: Dr. Qiu Haibo and Dr. Jianfeng Xie

Participating ICUs: Zhongda Hospital, Southeast University, Department of Critical Care Medicine: Dr Qiu Haibo, Dr Xie Jianfeng. Yijishan Hospital, First Affiliated Hospital of Wannan Medical College, Department of Critical Care

Medicine: Dr Lu Wei-Hua, Dr Wang Zhen. First Affiliate Hospital of Kunming Medical University, Micu/Eicu: Prof Chuanyun Qian, Dr Jili Luo. Qilu Hospital of Shandong University, Department of Critical Care Medicine: Dr Xiaomei Chen, Dr Hao Wang. Hebei Petrochina Central Hospital, Intensive Care Unit: Dr Peng Zhao, Dr Juan Zhao. Hangzhou Second Hospital, Affiliated Hospital of Hangzhou Normal University: Prof Qiu Wusi, Miss Chen Mingmin. Tianjin Third Central Hospital, Department of Critical Care Medicine: Dr Lei Xu, Dr Chengfen Yin. Shanghai General Hospital, Shanghai Jiao Tong University School of Medicine, Department Of Critical Care Medicine: Dr Ruilan Wang, Dr Jinfeng Wang. The Second Hospital of Jilin University, Department Of Critical Care: Dr Yongjie Yin, Dr Min Zhang. Taizhou People S Hospital, Intensive Care Unit: Dr Jilu Ye, Dr Chungfang Hu. The First Affiliated Hospital of Nanjing Medical University, Department Of Geriatrics Intensive Care Unit: Dr Suming Zhou, Dr Min Huang. Zhejiang Hospital, Intensive Care Unit: Prof Jing Yan, Dr Yan Wang. Henan Provincial People S Hospital, Department of Critical Care Medicine: Dr Bingyu Qin, Dr Ling Ye. Qingdao Municipal Hospital, Intensive Care Unit: Dr Xie Weifeng. The Second Hospital of Lanzhou University, Department Of Critical Care Medicine: Dr Li Peije, Dr Nan Geng.

Hong Kong

National Coordinator: Dr. Lowell Ling

Participating ICUs: The Chinese University of Hong Kong, Prince of Wales Hospital, Department Of Anaesthesia And Intensive Care: Dr Lowell Ling.

Japan

National Coordinator: Dr. Yoshiro Hayashi

Participating ICUs: Kameda Medical Center, Department of Intensive Care Medicine: Dr Yoshiro Hayashi, Dr Toshiyuki Karumai. University Hospital Kyoto Prefectural University of Medicine, Intensive Care Unit: Dr Masaki Yamasaki, Dr Satoru Hashimoto. Hiroshima University Hospital, ICU: Dr Koji Hosokawa. Yokosuka General Hospital Uwamachi, Critical Care Medicine: Dr Jun Makino. Tokyo Metropolitan Tama Medical Center, Emergency and Critical Care Center: Dr Takeo Matsuyoshi. Kurashiki Central Hospital, Emergency Intensive Care Unit: Dr Akira Kuriyama. Tokyo Medical and Dental University, Department of Intensive Care Medicine: Dr Hidenobu Shigemitsu, Dr Yuka Mishima, Dr Michio Nagashima. St. Marianna University School of Medicine Hospital, Mixed ICU: Dr Hideki Yoshida, Prof. Shigeki Fujitani. Osaka City General Hospital, Emergency and Critical Care Medical Hospital: Dr Koichiro Omori, Dr Hiroshi Rinka. St. Marianna University School of Medicine, Yokohama City Seibu Hospital, Mixed ICU: Dr Hiroki Saito, Dr Kaori Atobe. Yokohama City University Hospital, Infection Prevention and Control Department: Dr Hideaki Kato. Yokohama City University Hospital, Intensive Care Department: Dr Shunsuke Takaki.

Malaysia

National Coordinator: Dr. Helmi Sulaiman

Participating ICUs: University Malaya Medical Centre, Department of Anaesthesiology and Intensive Care: Dr M. Shahnaz Hasan, Dr Muhamad Fadhil Hadi Jamaluddin. Hospital Tengku Ampuan Rahimah, Anaesthesia and Intensive Care: Dr Lee See Pheng, Dr Sheshendrasurian Visvalingam. Hospital Sarikei, Anaesthesiology & Intensive Care Unit: Dr Mun Thing Liew, Dr Siong Ling Danny Wong. Queen Elizabeth 1 Hospital, Department of Anaesthesiology and Intensive Care: Dr Kean Khang Fong, Dr Hamizah Bt Abdul Rahman. Hospital Serdang, Cardiothoracic and Perfusion Unit: Dr Zuraini Md Noor, Dr Lee Kok Tong. Hospital Tuanku Fauziah, Intensive Care Unit: Dr Abd. Hamid Azman. School Of Medical Sciences Universiti Sains Malaysia, Department of Anaesthesiology and Intensive Care: Dr Mohd Zulfakar Mazlan. Hospital Universiti Sains Malaysia, Department of Anaesthesiology and Intensive Care: Dr Saedah Ali.

Philippines

National Coordinator: Dr. Aaron Mark Hernandez

Participating ICUs: The Medical City Ortigas, Intensive Care Unit: Dr Anton Abello.

Republic Of Korea

National Coordinator: Dr Kyeongman Jeon

Participating ICUs: Samsung Medical Center, Medical Icu: Dr Kyeongman Jeon. Seoul National University Hospital, Medical Icu: Dr Sang-Min Lee. Hallym University Sacred Heart Hospital, Micu: Dr Sunghoon Park. Micu, Chonbuk National University Hospital: Prof Dr Seung Yong Park. Seoul National University Bundang Hospital, Medical Icu: Dr Sung Yoon Lim.

Singapore

National Coordinator: A/Prof Andrea Lay Hoon Kwa, Dr Qing Yuan Goh

Participating ICUs: Singapore General Hospital, Surgical Intensive Care Unit: Dr Qing Yuan Goh, A/Prof Shin Yi Ng. Singapore General Hospital, Neurosurgical Intensive Care Unit: Dr Sui An Lie, A/Prof Andrea Lay Hoon Kwa. Singapore General Hospital, Medical Intensive Care Unit: Dr Ken Junyang Goh. National University Hospital System Medical Intensive Care Unit: Dr Andrew Yunkai Li. Tan Tock Seng Hospital, Surgical Intensive Care Unit, Neurological Intensive Care Unit: Adj Asst Prof Caroline Yu Ming Ong, Dr Jia Yan Lim. Changi General Hospital, Medical Intensive Care Unit: Dr Jessica Lishan Quah, Dr Kangqi Ng. Changi General Hospital, Surgical Intensive Care Unit: Dr Louis Xiang Long Ng.

Taiwan

National Coordinator: Dr. Tony Yu-Chang Yeh

Participating ICUs: National Taiwan University Hospital, Sicu: Dr Yu Chang Yeh, Dr Nai-Kuan Chou. National Cheng Kung University Hospital, Division of Critical Care Medicine, Department of Internal Medicine: Dr Cong-Tat Cia. Mackay Memorial Hospital, Department of Critical Care Medicine: Dr Ting-Yu Hu, Dr Li-Kuo Kuo. National Taiwan University Hospital, Department of Internal Medicine, Micu: Dr Shih-Chi Ku.

Thailand

National Coordinator: Prof (Associate) Phunsup Wongsurakiat

Participating ICUs: Siriraj Hospital, Mahidol University, Critical Respitarory Care Unit, Department of Medicine: Prof (Associate) Phunsup Wongsurakiat. Vajira Hospital, Department of Internal Medicine: Dr Yutthana Apichatbutr, Dr Supattra Chiewroongroj.

Middle East and North Africa

Dubai

National Coordinator: Dr. Adel Alsisi

Participating ICUs: Dubai Hospital, Icu Department: Dr Rashid Nadeem, Dr Ashraf El Houfi.

Egypt

National Coordinator: Dr. Adel Alsisi

Participating ICUs: Cairo University Hospital (Qasr Al Ainy), Critical Care Department: Dr Adel Alsisi, Dr Amr Elhadidy, Dr Mina Barsoum.Medical Research Institute, Alexandria University, Biomedical Informatics and Medical Statistics (Icu): Dr Nermin Osman. Tanta University Hospital, Anaesthesia and Critical Care Department: Dr Tarek Mostafa. Tanta University Faculty of Medicine, Emergency Medicine and Traumatology Department: Dr Mohamed Elbahnasawy. Tanta University Emergency Hospital, Emergency, And Traumatology Department Critical Care Unit: Dr Ahmed Saber. Nasr City Health Insurance Hospital, Medical Icu: Dr Amer Aldhalia. Wingat Royal Hospital, Wingat Icu: Dr Omar Elmandouh. Elsahel Teaching Hospital, Icu: Dr Ahmed Elsayed. Ain Shams University Hospitals, Department of General Surgery: Dr Merihan A. Elbadawy, Dr Ahmed K. Awad. Alexandria Faculty of Medicine, Dialysis Intensive Care Unit: Miss Hanan M. Hemead.

Iran

National Coordinator: Prof. Farid Zand

Participating ICUs: Shiraz University of Medical Sciences, Anesthesiology and Critical Care Research Center: Prof Farid Zand, Dr Maryam Ouhadian. Ahvaz Jundishapur University of Medical Sciences, Air Pollution and Respiratory Diseases Research Center: Dr Seyed Hamid Borsi,, Dr Zahra Mehraban. Ahvaz Jundishapur University of Medical Sciences, Neurology Department: Dr Davood Kashipazha. Ahvaz Jundishapur University of Medical Sciences, Infectious and Tropical Diseases Research Center, Health Research Institute: Dr Fatemeh Ahmadi. Ahvaz Jundishapur

University of Medical, Pain Research Center: Dr Mohsen Savaie, Dr Farhad Soltani, Dr Mahboobeh Rashidi, Dr Reza Baghbanian, Dr Fatemeh Javaherforoosh, Dr Fereshteh Amiri. Ahvaz Jundishapur University of Medical Sciences, Neurosurgery Department, Dr Arash Kiani. Ahvaz Jundishapur University of Medical Sciences, General Surgery Department, Dr Mohammad Amin Zargar. Tabriz University of Medical Sciences, Research Center for Integrative Medicine in Aging, Aging Research Institute: Prof Ata Mahmoodpoor. Jahrom University of Medical Sciences, Peimanieh Hospital : Dr Fatemeh Aalinezhad. Shiraz University of Medical Sciences, Shahid Rajaee Trauma Hospital : Dr Gholamreza Dabiri. Shiraz University of Medical Sciences, Trauma Research Center, Shahid Rajaee Hospital : Dr Golnar Sabetian, Dr Hakimeh Sarshad. Shiraz University of Medical Sciences, Anesthesiology and Critical Care Research Center: Dr Mansoor Masjedi, Dr Ramin Tajvidi. Zahedan University of Medical Sciences, Anesthesiology and Critical Care Department: Dr Seyed Mohammad Nasirodin (S.M.N.) Tabatabaei.

Iraq

Participating ICUs: Ibn Zuhur Hospital, Icu: Dr Abdullah Khudhur Ahmed.

Israel

National Coordinator: Prof. Pierre Singer

Participating ICUs: Rabin Medical Center Beilinson Hospital, General Intensive Care: Prof Pierre Singer, Dr Ilya Kagan, Dr Merav Rigler. Shaare Zedek Medical Center, Intensive Care Unit: Dr Daniel Belman, Dr Phillip Levin.

Jordan

Participating ICUs: Abdali Hospital, Icu: Dr Belal Harara, Dr Adei Diab.

Lebanon

National Coordinator: Dr Fayez Abillama

Participating ICUs: Lebanese American University Medical Center Rizk Hospital, Intensive Care: Dr Fayez Abillama, Dr Rebecca Ibrahim, Dr Aya Fares.

Libya

National Coordinator: Dr. Muhammed Elhadi

Participating ICUs: Aljalla Benghazi Center, Micu: Dr Ahmad Buimsaedah. Almokhtar Clinic, Intensive Care Unit: Dr Marwa Gamra. Althawra Central Hospital, Intensive Care Unit: Dr Ahmed Aqeelah. Brega General Hospital Bgh Libya, Icu: Dr Almajdoub Ali Mohammed Ali, Dr Ahmed Gaber Sadik Homaidan. National Heart Institute, Micu: Dr Bushray Almiqlash, Dr Hala Bilkhayr. Tobruk Medical Centre, Medical Icu: Dr Ahmad Bouhuwaish, Dr Ahmed Sa Taher. Tripoli Central Hospital, Icu: Dr Eman Abdulwahed, Dr Fathi A Abousnina, Dr Aisha Khaled Hdada. Tripoli Central Hospital, Unit C: Dr Rania Jobran. Zliten Medical Center, Icu of Zliten Medical Center: Dr Hayat Ben Hasan, Dr Rabab Shaban Ben Hasan.

Morocco

National Coordinator: Prof. Khalid Abidi

Participating ICUs: Avicenne Military Hospital, Icu: Dr Issam Serghini, Pr Rachid Seddiki. CHU Hassan II Fès, Intensive Care Unit A4: Dr Brahim Boukatta, Dr Nabil Kanjaa. Hospital Of Specialties, Critical Care Unit of Neurology and Neurosurgery: Prof Doumiri Mouhssine, Prof Maazouzi Ahmed Wajdi. Ibn Sina University Hospital, faculty of Medicine and Pharmacy, Mohammed V University in Rabat, Medical Icu: Pr Tarek Dendane, Pr Amine Ali Zeggwagh. Mohammed VI University Hospital of Oujda, Faculty Of Medicine and Pharmacy Oujda, Mohammed Premier University, Anesthesia and Resuscitation Department: Prof Brahim Housni, Dr Oujidi Younes. Mohammed VI University Hospital, Medical Icu, Marrakech: Prof Abdelhamid Hachimi. National Institute of Oncology of Rabat, Intensive Care Unit: Prof A Ghannam, Prof Z Belkhadir.

Palestine

Participating ICUs: ICU, Alia governmental hospital, Hebron / West Bank, Palestine: Dr. Sarah Amro. Gaza city, Alshifaa hospital, Gaza, Palestine: DR. Mustafa Abu Jayyab.

Qatar

National Coordinator: Dr Ali Aithssain

Participating ICUs: Hamad General Hospital, Medical Icu: Dr Ali Ait Hssain, Dr Abdurahaman Elbuzidi. Al Wakrah Hospital, Critical Care: Dr Edin Karic. Hamad General Hospital, Sicu: Dr Marcus Lance, Dr Shaikh Nissar.

Saudi Arabia

Participating ICUs: King Faisal Specialist Hospital &Research Center, Adult Critical Care Medicine: Dr Hend Sallam. Prince Sultan Medical Military Center, Intensive Care Unit: Dr Omar Elrabi, Dr Ghaleb A Almekhlafi. Security Force Hospital - Riyadh, Critical Care Unit: Dr Maher Awad, Dr Ahmed Aljabbary.

Syria

Participating ICUs: Al Mouwasat University Hospital, Icu: Dr Mohammad Karam Chaaban. Assad University Hospital, Neurological Intensive Care Unit: Dr Natalia Abu-Sayf. Damascus University Cardiac Surgery Hospital Near Al-Mouwasat University Hospital, Mazzeh Kiwan, Cardiac Surgery Icu: Dr Mohammad Al-Jadaan, Miss Lubna Bakr.

Tunisia

National Coordinator: Dr Mounir Bouaziz

Participating ICUs: Habib Bourguiba University Hospital, Department of Intensive Care: Dr Mounir Bouaziz, Dr Olfa Turki. Military Hospital of Tunis, Department of Anesthesiology And Intensive Care Unit, Lr12dn01: Pr Walid Sellami.

Latin America and The Caribbean

Argentina

National Coordinator: Dr. Gabriela Vidal

Participating ICUs: Hcas Cuenca Alta, Terapia Intensiva: Dr Pablo Centeno, Lic Natalia Morvillo. Hospital Central De Formosa, Servicio De Terapia Intensiva: Dr José Oscar Acevedo, Dr Patricia Mabel Lopez. Hospital Español De Mendoza, Terapia Intensiva De Adultos: Dr Rubén Fernández, Dr Matías Segura. Hospital Zatti, Ucia: Dra Marta Aparicio, Microbiologa Irene Alonzo. Instituto De Diagnostico De La Plata, Unidad De Terapia Intensiva: Dr Yanina Nuccetelli, Dr Pablo Montefiore.

Colombia

National Coordinator: Mario Arias

Participating ICUs: Clinica Universidad De La Sabana, Critical Care Unit : Dr Luis Felipe Reyes. Universidad De La Sabana, Infectious Diseases Department: Dr Luis Felipe Reyes.

Mexico

National Coordinator: Dr Silvio A. Ñamendys-Silva

Participating ICUs: Hospital Medica Sur, Department of Critical Care Medicine: Dr Silvio A. Ñamendys-Silva, Dr Juan P. Romero-Gonzalez. Centenario Hospital Miguel Hidalgo, Centenario Hospital Miguel Hidalgo: Dr Mariana Hermosillo, Dr Roberto Alejandro Castillo. Hospital General De Zona 14, Intensive Care Unit: Dr Jesús Nicolás Pantoja Leal, Dr Candy Garcia Aguilar. Hospital General Regional No.1, IMSS Tlaxcala: Dr Mara Ocotlan Gonzalez Herrera, Dr Missael Vladimir Espinoza Villafuerte. Hospital H+ Queretaro, Unidad De Terapia Intensiva Adultos: Dr Manuel Lomeli-Teran. Instituto Nacional de Ciencias Medicas y Nutricion Salvador Zubiran, Division of Pulmonary, Anesthesia and Critical Care Medicine: Dr Jose G. Dominguez-Cherit, Dr Adrian Davalos-Alvarez, Dr Silvio A. Ñamendys-Silva. UMAE Hospital de Especialidades Antonio Fraga Mouret, Centro Médico Nacional La RazaIMSS, Terapia Intensiva Hospital de Especialidades CMN La Raza: Dr Luis Sánchez-Hurtado, Dr Brigitte Tejeda-Huezo. Hospital General San Juan del Rio, Querétaro, , Unidad de Terapia Intensiva de Adultos: Dr Orlando R Perez-Nieto, Dr Ernesto Deloya Tomas.

Europe And Central Asia

Belgium

National Coordinator: Dr. Liesbet De Bus

Scientific Committee: Prof. Jan De Waele

Recruitment of participating ICUs worldwide: Mr. Guy Francois

Participating ICUs: Ghent University Hospital, Intensive Care Unit: Dr Liesbet De Bus, Dr Jan De Waele. A.S.Z., Iz: Dr Isabelle Hollevoet. Az Nikolaas, Icu: Dr Wouter Denys. Az Sint-Jan Av Brugge - Oostende Campus Brugge, Icu: Dr Marc Bourgeois. Az Sint-Lucas, Department of Intensive Care: Dr Sofie F.M. Vanderhaeghen. Centre Hospitalier De Jolimont, Soins Intensifs : Dr Jean-Baptiste Mesland, Dr Pierre Henin. Chu Ambroise Paré, Unité Des Soins Intensifs : Dr Lionel Haentjens. Chu Charleroi, Medico-Surgical Icu: Dr Patrick Biston, Mrs Cindérella Noel. Chu Liège, Soins Intensifs : Dr Nathalie Layos, Dr Benoît Misset. Clinique Saint-Pierre, Intensive Care Unit : Dr Nicolas De Schryver, Dr Nicolas Serck. Cliniques Universitaires Saint-Luc, UCLouvain, Soins Intensifs : Dr Xavier Wittebole. Uzbrussel, Intensieve Zorgen: Prof Elisabeth De Waele, Mrs Godelive Opdenacker.

Bosnia And Herzegovina

National Coordinator: Dr Pedja Kovacevic

Participating ICUs: University Clinical Centre of The Republic Of Srpska, Medical Intensive Care Unit: Dr Pedja Kovacevic, Dr Biljana Zlojutro.

Croatia

National Coordinator: Dr Ina Filipovic-Grcic

Participating ICUs: General Hospital Dubrovnik, Anesthesiology, And Intensive Care: Dr Aida Custovic, Dr Ina Filipovic-Grcic. University Hospital Centre Zagreb, Medical Intensive Care Unit: Prof Radovan Radonic, Dr Ana Vujaklija Brajkovic. University Hospital Dubrava, Clinical Department of Anesthesiology, Reanimatology and Intensive Care: Prof Jasminka Persec, Dr Sanja Sakan, Dr Mario Nikolic, Dr Hrvoje Lasic.

France

National Coordinator: Prof. Marc Leone

Scientific Committee: Prof. Jean-François Timsit, Prof. Etienne Ruppe, Mr. Stephane Ruckly, Prof. Philippe Montravers

Participating ICUs: Hôpital Nord, Réanimation Polyvalente et Traumatologique : Pr Marc Leone, Dr Charlotte Arbelot. Bichat Claude Bernard, Réanimation Médicale et Infectieuse : Prof Jean-François Timsit, Mme Juliette Patrier. Bichat-Claude Bernard Hospital, Ap-Hp, Anesthesiology And Critical Care Medicine Department, Dmu Parabol: Dr Nathalie Zappella, Pr P. Montravers. Centre Hospitalier De Bigorre, Service De Réanimation Polyvalente : Dr Thierry Dulac, Dr Jérémy Castanera. Centre Hospitalier De Cholet, Réanimation Polyvalente : Dr Johann Auchabie, Dr Anthony Le Meur. Centre Hospitalier De Dieppe, Médecine Intensive Réanimation : Dr A. Marchalot, Dr M. Beuzelin. Centre Hospitalier De Pau, Réanimation Polyvalente : Dr Alexandre Massri, Dr Charlotte Guesdon. Ch Annecy Genevois, Réanimation Polyvalente : Dr Etienne Escudier. Ch De Charleville-Mézières, Médecine Intensive Réanimation : Dr Philippe Mateu, Dr Jérémy Rosman. Ch Tourcoing, Service De Reanimation: Dr Olivier

Leroy, Dr Serge Alfandari. Chu Compiegne Noyon, Réanimation : Dr Alexandru Nica. Chu Gabriel Montpied, Médecine Intensive Et Réanimation : Dr Bertrand Souweine, Dr Elisabeth Coupez. Chu Lille, Hôpital Roger Salengro, Pôle De Réanimation : Dr Thibault Duburcq. Chu Lille, Surgical Critical Care, Department of Anesthesiology and Critical Care: Prof Eric Kipnis, Dr Perrine Bortolotti. Chu Rennes, Service De Maladies Infectieuses Et Réanimation Médicale : Dr Mathieu Le Souhaitier. Cochin, Medecine Intensive Reanimation: Dr Jean-Paul Mira. Ghef Site De Marne-La-Vallée, Réanimation Polyvalente : Dr Pierre Garcon, Dr Matthieu Duprey. Groupe Hospitalier Nord Essonne - Site Longjumeau, Réanimation Polyvalente : Dr Martial Thyrault, Dr Rémi Paulet. Groupe Hospitalier Paris Saint Joseph, Médecine Intensive et Réanimation : Dr François Philippart, Dr Marc Tran, Dr Cédric Bruel. Hôpital Beaujon, Department of Anesthesiology and Critical Care: Dr Emmanuel Weiss, Dr Sylvie Janny, Dr Arnaud Foucrier. Hopital De Gui De Chauliac, Departement Anesthesie Reanimation Gui De Chauliac : Dr Pierre-François Perrigault, Dr Flora Djanikian. Hôpital De La Source, Centre Hospitalier Régional D'orléans, Médecine Intensive & Réanimation (Medical Icu): Dr François Barbier. Hôpital De La Timone, Médecine Intensive Réanimation : Dr Marc Gainnier, Dr Jérémy Bourenne. Hopital De Mercy, Chr Metz-Thionville, Service De Réanimation Polyvalente Et Usc: Dr Guillaume Louis. Hopital Du Scorff, Service De Réanimation : Dr Roland Smonig. Hôpital Edouard Herriot, Médecine Intensive-Réanimation : Dr Laurent Argaud, Dr Thomas Baudry. Hôpital Henri Mondor, Service De Réanimation Médicale : Pr Armand Mekonted Dessap, Dr Keyvan Razazi. Hôpital Louis Pasteur, Réanimation : Dr Pierre Kalfon, Mr Gaëtan Badre. Montpellier University Hospital, Intensive Care Medicine Lapeyronie Hospital: Dr Romaric Larcher. Nimes University Hospital, Service Des Réanimations : Prof Jean-Yves Lefrant, Dr Claire Roger. Purpan, Réanimation Polyvalente : Dr Benjamine Sarton, Dr Stein Silva. Sorbonne Universite Pitie Salpetriere, Médecine Intensive Et Réanimation Neurologique : Dr Sophie Demeret, Dr Loïc Le Guennec. Sud Essonne Hospital, Department of Intensive Care Medicine: Dr Shidasp Siami, Mrs Christelle Aparicio. Tenon Hospital, Service De Médecine Intensive Réanimation : Dr Guillaume Voiriot, Dr Muriel Fartoukh. University Hospital Of Poitiers, Surgical And Neuro Intensive Care Units: Dr Claire Dahyot-Fizelier, Dr Nadia Imzi. University Of Montpellier, Phymedexp Inserm Cnrs: Dr Kada Klouche.

Germany

National Coordinator: Prof. Hendrik Bracht

Participating ICUs: University Hospital Ulm, Icu G1: Dr Hendrik Bracht, Dr Sandra Hoheisen. Jena University Hospital, Dept. Of Anesthesiology and Intensive Care Medicine: Dr Frank Bloos, Dr Daniel Thomas-Rueddel. Universitätsklinikum Leipzig, Medical Icu: Dr Sirak Petros, Dr Bastian Pasieka. University Hospital Heidelberg, Station 13 Iopsis: Dr Simon Dubler, Dr Karsten Schmidt. University Hospital Muenster, Department of Anesthesiology, Intensive Care Medicine and Pain Therapy: Dr Antje Gottschalk, Dr Carola Wempe. University

Hospital of Saarland, Dept. Of Internal Medicine V - Pneumology, Allergology and Critical Care Medicine: Prof Philippe Lepper, Dr Carlos Metz.

Kazakhstan

National Coordinator: Dr. Dmitriy Viderman

Participating ICUs: University Medical Center, National Research Oncology Center, Intensive Care Unit: Dr Dmitriy Viderman, Dr. Yerlan Umbetzhanov. Karaganda Medical University, Department of Emergency Medicine, Anesthesiology and Resuscitation Non-Commercial Joint-Stock Company: Associate Prof., Dr Miras Mugazov, Dr Yelena Bazhykayeva. Medical Center Hospital of The President's Affairs Administration of The Republic of Kazakhstan, Intensive Care Unit: Dr Zhannur Kaligozhin, Dr Baurzhan Babashev. National Research Oncology Center, Department of Oncohematological Resuscitation, Resuscitation, Intensive Care: Dr Yevgeniy Merenkov, Dr Talgat Temirov.

Greece

National Coordinator: Dr. Kostoula Arvaniti

Participating ICUs: Papageorgiou Hospital, Intensive Care Unit: Dr Kostoula Arvaniti, Dr Dimitrios Smyrniotis. Agioi Anargiroi Hospital, Agioi Anargiroi Icu: Dr Vasiliki Psallida, Dr Georgios Fildisis. G Papanikolaou General Hospital, 1st Icu: Dr Vasiliki Soulountsi, Dr Evangelos Kaimakamis. G Papanikolaou General Hospital, B Icu: Dr Cristina Iasonidou, Dr Sofia Papoti. General Hospital G. Gennhmatas, Gnth "G Gennhmatas": Dr Foteini Renta, Dr Maria Vasileiou. General Hospital of Athens G. Gennimatas, Icu: Dr Vasiliki Romanou, Dr Vasiliki Koutsoukou. Gh Imathia Veria, Icu: Dr Mariana Kristina Matei, Dr Leora Moldovan. Icu, Hygeia General Hospital: Dr Ilias Karaiskos, Dr Harry Paskalis. Intensive Care Unit, General Hospital of Giannitsa: Dr Kyriaki Marmanidou. Intensive Care Unit, Hippocration General Hospital Of Athens: Dr M. Papanikolaou, Dr C.Kampolis. Katerini General Hospital, Gnk Icu: Dr Marina Oikonomou, Dr Evangelos Kogkopoulos. Konstantopoulion-Patision Hospital, Icu: Dr Charikleia Nikolaou, Dr Anastasios Sakkalis. Mediterraneo Hospital, Icu/Hdu : Dr Marinos Chatzis, Dr Maria Georgopoulou. Saint Savvas Hospital, Icu: Dr Anna Efthymiou, Dr Vasiliki Chantziara. Sismanogleio Hospital, Sismanogleion Icu: Dr Aikaterini Sakagianni, Dr Zoi (Zoe) Athanasa (Athanassa). Theageneio Anticancer Hospital, Icu: Dr Eirini Papageorgiou, Dr Fadi Ali. University Hospital Attikon, National And Kapodistrian University Of Athens, Department Of Critical Care: Pr Georges Dimopoulos, Dr Mariota Panagiota Almiroudi. University Hospital Heraklion, Department of Intensive Care: Dr Polychronis Malliotakis, Dr Diamantina Marouli. University Hospital of Alexandroupolis, Department Of Intensive Care: Dr Vasiliki Theodorou, Dr Ioannis Retselas. University Hospital of Ioannina, Intensive Care Unit: Pr Vasilios Kouroulas, A/Pr Georgios Papathanakos.

Italy

National Coordinator: Prof. Matteo Bassetti and Prof. Daniele Roberto Giacobbe

Participating ICUs: Città Della Salute E Della Scienza - Molinette, Anestesia E Rianimazione Universitaria: Dr Giorgia Montrucchio, Dr Gabriele Sales. Fondazione Policlinico Universitario A. Gemelli Irccs. Universita Cattolica Del Sacro Cuore. Italy, Uoc Di Anestesia, Rianimazione, Terpia Intensiva E Tossicologia Clinica: Dr Gennaro De Pascale, Dr Luca Maria Montini, Dr Simone Carelli, Dr Joel Vargas, Ms Valentina Di Gravio. Irccs Ospedale Policlinico San Martino, U.O. Anestesia E Rianimazione: Prof Daniele Roberto Giacobbe, Dr Angelo Gratarola, Dr Elisa Porcile, Dr Michele Mirabella. Irccs Sacro Cuore Don Calabria, Terapia Intensiva: Dr Ivan Daroui, Dr Giovanni Lodi. Madonna Delle Grazie, U.O.C. Anestesia E Rianimazione: Dr Francesco Zuccaro, Dr Maria Grazia Schlevenin. Ospedale Policlinico San Martino, Irccs Per L'oncologia E Le Neuroscienze, Uo Clinica Anestesiologica E Terapia Intensiva: Prof Paolo Pelosi, Dr Denise Battaglini. Policlino Paolo Giaccone, Università Degli Studi Di Palermo, Terapia Intensiva Polivalente: Dr Andrea Cortegiani, Dr Mariachiara Ippolito, Dr Davide Bellina, Dr Andrea Di Guardo. Regina Elena National Cancer Institute of Rome, Anesthesia and Intensive Care Department: Dr Lorella Pelagalli, Dr Marco Covotta. Sant'andrea Hospital Sapienza University of Rome, Department of Medical And Surgical Science And Translational Medicine Intensive Care Unit: Dr Monica Rocco, Dr Silvia Fiorelli. University Hospital O.O.R.R., Department of Anesthesia And Intensive Care: Prof Antonella Cotoia, Dr Anna Chiara Rizzo.

Poland

National Coordinator: Dr Adam Mikstacki

Participating ICUs: Hospital In Puszczykowo, Poznan University of Medical Sciences, Department of Anaesthesiology and Intensive Therapy: Dr Adam Mikstacki, Dr Barbara Tamowicz. 10 Wojskowy Szpital Kliniczny, Oddzial Kliniczny Anestezjologii I Intensywnej Terapii: Dr Irmina Kaptur Komorowska, Dr Anna Szczesniak. Szpital Wojewodzki W Opolu, Oddzial Anestezjologii I Intensywnej Terapii: Dr Jozef Bojko, Dr Anna Kotkowska. Uck Wum, Oddzial Intensywnej Terapii (Icu): Dr Paulina Walczak-Wieteska, Dr Dominika Wasowska. Wojewodzki Szpital Zespolony, Oddzial Anestezjologii I Intensywnej Terapii: Dr Tomasz Nowakowski, Dr Hanna Broda. Wss Im. Wl. Bieganskiego, Oddzial Anestezjologii I Intensywnej Terapii - Osrodek Pozaustrojowych Technik Wspomagania Czynnosci Nerek I Wątroby: Prof Assoc Mariusz Peichota, Dr Iwona Pietraszek-Grzywaczewska.

Republic Of Ireland

National Coordinator: Prof Ignacio Martin-Loeches

Participating ICUs: St Jame's Hospital, Intensive Care Unit: Prof Ignacio Martin-Loeches, Dr Alessandra Bisanti.

Portugal

National Coordinator: Prof. José Artur Paiva

Scientific Committee: Prof. Pedro Póvoa

Participating Icus: Centro Hospitalar Medio Tejo - Unidade Abrantes, Ucip: Dr Nuno Cartoze, Dr Tiago Pereira. Centro Hospitalar Universitário do Porto, Sci 1: Dr Nádia Guimarães, Dr Madalena Alves. Centro Hospitalar Vila Nova De Gaia/Espinho, Unidade De Cuidados Intensivos Polivalente: Dr Ana Josefina Pinheiro Marques, Dr Ana Rios Pinto. CHUA Faro, Smi-1 : Dr Andriy Krystopchuk, Dr Ana Teresa. Hospital De Cascais Dr Jose De Almeida, Unidade de Cuidados Intensivos: Dr António Manuel Pereira de Figueiredo, Dr Isabel Botelho. Hospital Curry Cabral, Intensive Care Medicine Department: Dr Tiago Duarte. Hospital Sao Francisco Xavier, CHLO, Unidade De Cuidados Intensivos Polivalente: Dr Vasco Costa, Dr Rui Pedro Cunha. Hospital Pedro Hispano, Serviço De Medicina Intensiva: Dr Elena Molinos, Dr Tito da Costa. CHULC, Hospital Sao José, Unidade de Urgência Médica: Dr Sara Ledo, Dr Joana Queiró. ULS Litoral Alentejano, Serviço de Medicina Intensiva: Dr Dulce Pascoalinho. ULS Nordeste, Unidade de Cuidados Intensivost: Dr Cristina Nunes. ULSAM, UCI: Dr José Pedro Moura, Dr Énio Pereira. ULS Baixo Alentejo, Unidade Cuidados Intensivos Polivalente: Dr António Carvalho Mendes.

Romania

National Coordinator: Dr Liana Valeanu

Participating ICUs: Emergency Institute for Cardiovascular Diseases Prof. Dr. C. C. Iliescu, 1st Anesthesia and Intensive Care Department: Dr Liana Valeanu, Prof Serban Bubenek-Turconi. Clinical Emergency Hospital Bucharest, Anesthesia and Intensive Care Department: Prof Ioana Marina Grintescu, Dr Cristian Cobilinschi. Emergency Institute for Cardiovascular Diseases Prof. Dr. C. C. Iliescu, 2nd Anesthesia and Intensive Care Department: Prof Daniela Carmen Filipescu, Dr Cornelia Elena Predoi. Fundeni Clinical Institute, 3rd Department of Anesthesia and Intensive Care: Prof Dana Tomescu, Dr Mihai Popescu, Dr Alexandra Marcu. University Of Medicine and Pharmacy "Grigore T Popa", Anesthesia and Intensive Care Department: Prof Ioana Grigoras, Dr Olguta Lungu.

Russian Federation

National Coordinator: Prof. Alexey Gritsan

Participating ICUs: V.F. Voino-Yasenetsky Krasnoyarsk State Medical University, Krasnoyarsk Regional Clinical Hospital, Dep. Anaesthesiology and Intensive Care #3: Prof Alexey Gritsan. City Clinical N.I.Pirogov Hospital, Clinical Pharmacology: Dr Anastasia Anderzhanova, Dr Yulia Meleshkina. City Clinical N.I.Pirogov Hospital, Icu: Dr Marat Magomedov. E.A. Vagner Perm State Medical University, Intensive Care Unit: Prof Nadezhda Zubareva, Dr Maksim Tribulev. Krasnoyarsk Regional Clinical Hospital, Dep. Anaesthesiology and Intensive Care #3: Dr Denis Gaigolnik. Petrovsky National Research Centre of Surgery, Intensive Care: Dr Aleksandr Eremenko, Dr Natala Vistovskaya, Dr Maria Chukina. Privolzhskiy District Medical Center, Department Anesthesiology and Intensive Care: Dr Vladislav Belskiy, Dr Mikhail Furman.

Spain

National Coordinator: Dr. Ricard Ferrer Rocca

Participating ICUs: Vall D'herbon, Intensive Care Medicine: Dr Ricard Ferrer Rocca, Dr Maria Martinez, Dr Vanessa Casares. Hospital Clinic De Barcelona, Surgical Icu: Dr Ricard Mellado Artigas. Hospital De La Santa Creu I Sant Pau, Intensive Care Unit : Dr Paula Vera, Dr Matias Flores. Hospital De Terrassa, Medicina Intensiva: Dr Joaquin Amador Amerigo. Hospital Del Mar, Critical Care Unit: Dr Maria Pilar Gracia Arnillas, Dr Rosana Munoz Bermudez. Hospital Germans Trias I Pujol, Critical Care Unit: Prof, Dr Fernando Armestar, Dr Beatriz Catalan, Dr Regina Roig, Dr Laura Raguer, Dr María Dolores Quesada. Hospital Parc Tauli, Icu: Dr Emilio Diaz Santos, Dr Gemma Gomà. Hospital Punta De Europa, Intensive Care Unit : Dr Alejandro Ubeda, Dra Maria Salgado. Hospital Universitario Central De Asturia, Uci-Huca: Dr Lorena Forcelledo Espina, Dr Emilio Garcia Prieto. Hospital Universitario La Paz, Intensive Care Unit, Servicio De Medicina Intensiva: Dra Mj Asensio, Dra M. Rodriguez. Hospital Universitario La Paz, Surgical Critical Care Unit : Dr Emilio Maseda, Dr Alejandro Suarez De La Rica. Hospital Universitarion Son Espases, Unidad De Cuidados Intensivos: Dr J Ignacio Ayestaran, Dr Mariana Novo. University Hospital Severo Ochoa, Intensive Care Unit: Dr Miguel Angel Blasco-Navalpotro, Dr Alberto Orejas Gallego.

Sweden

National Coordinator: Dr Fredrik Sjovall

Participating ICUs: Skane University Hospital, Intensive- And Perioperative Care: Dr Fredrik Sjövall, Dr Dzana Spahic. Ostra Sjukhuset Sahlgrenska University Hospital, Anopiva: Dr Carl Johan Svensson. Umeå University, Anesthesiology and Intensive Care Medicine, Surgical and Perioperative Sciences: Dr Michael Haney, Dr Alicia Edin. Universitetssjukhuset I Linkoping, Anopiva: Dr Joyce Åkerlund, Dr Lina De Geer.

Switzerland

National Coordinator: Dr. Josef Prazak

Scientific Committee: Dr. Niccolò Buetti

Participating ICUs: Inselspital, Bern University Hospital, Department of Intensive Care Medicine: Dr Josef Prazak, Dr Stephan Jakob. Chuv, Service De Médecine Intensive Adulte : Dr Jl Pagani, Mrs S Abed-Maillard.

Turkey

National Coordinator: Prof. Murat Akova, Dr. Abdullah Tarık Aslan

Participating ICUs: Hacettepe University of Faculty of Medicine, Intensive Care Unit(ICU): Dr Murat Akova, Dr Abdullah Tarik Aslan, Abdurrahman Yurtaslan Ankara Oncology Training and Research Hospital, Department of Anesthesiology: Dr Arif Timuroglu. Acibadem Fulya Hospital, Infectious Diseases: Dr Sesin Kocagoz, Dr Hulya Kusoglu. Acibadem Kadikoy Hospital, ICU: Dr Selcuk Mehtap, Dr Solakoğlu Ceyhun. Ankara University Faculty Medicine Ibni Sina Hospital, Medical ICU: Prof. Dr. Neriman Defne Altintas, Dr Leyla Talan. Ankara Yildirim

Beyazit University, Ankara City Hospital, Infectious Diseases and Clinical Microbiology: Dr Bircan Kayaaslan, Dr Ayşe Kaya Kalem. Aydin Adnan Menderes University Research Hospital, Anesthesia and Reanimation ICU: Prof. Dr. Ibrahim Kurt, Dr (Professor) Murat Telli, Dr (Associate Professor) Barcin Ozturk. Baskent University Hospital, Infectious Diseases and Clinical Microbiology: Dr Çiğdem Erol. Bitlis Goverment Central Hospital, Bitlis Icu: Dr Emine Kubra Dindar Demiray, Dr Sait Çolak. Duzce University Hospital, Medical ICU: Dr Türkay Akbas. Erciyes University, ICU: Prof. Dr. Kursat Gundogan, Dr Ali Sari. Fatih Sultan Mehmet Research and Training Hospital, Infection Diseases: Dr Canan Agalar, Dr Onur Çolak. Hitit University Erol Olcok Education and Research Hospital, Infectious Diseases and Clinical Microbiology: Prof. Dr. Nurcan (N) Baykam, Assistant Prof. Dr Ozlem (O) Akdogan. Istanbul Medipol University, Kosuyolu Hospital, Infectious Diseases and Clinical Microbiology: Dr Mesut Yilmaz, Dr Burcu Tunay, Dr Rumeysa Cakmak. Istanbul University-Cerrahpasa, Cerrahpasa Medical Faculty, Sadi Sun ICU: Prof.Dr. Nese Saltoglu, Ass Prof.Dr. Ridvan Karaali. Karadeniz Technical University Faculty of Medicine, Infectious Disease and Clinical Microbiology: Prof Dr. Iftihar Koksal, Assist. Prof. Firdevs Aksoy. Karadeniz Technical University Farabi Hospital, Anesthesia ICU 1: Dr Ahmet Eroglu. Kartal Dr. Lutfi Kirdar Training and Research Hospital, ICU: Dr Kemal Tolga Saracoglu, Dr Yeliz Bilir. Kayseri City Hospital, ICU: Dr Seda Guzeldag. Mersin University Hospital, Department of Infectious Diseases and Clinical Microbiology: Dr Gulden Ersoz, Dr Guliz Evik. Pamukkale Univ, Anesthesiology and Reanimation: Prof Hulya Sungurtekin, Dr Cansu Ozgen. School Of Medicine, Medipol Mega University Hospitals Complex, Department of Anesthesiology and Reanimation: Dr Cem Erdoğan. University of Health Sciences Diskapi Yildirim Beyazit Training and Research Hospital, The Department of Infectious Diseases and Clinical Microbiology and ICU: Dr Yunus Gürbüz, Dr Nilgün Altin. Turgut Ozal Medical Center, Department of Infectious Diseases and Clinical Microbiology: Dr Yasar Bayindir, Dr Yasemin Ersoy. University of Health Sciences Istanbul Umraniye Training and Research Hospital, Anaestesia and Reanimation: Dr Senay Goksu, Dr Ahmet Akyol. University of Health Sciences, Kartal Dr. Lutfi Kirdar Training and Research Hospital, Infectious Diseases and Clinical Microbiology: Prof Ayse Batirel, Dr Sabahat Cagan Aktas.

The United Kingdom

National Coordinator: Dr. Andrew Conway Morris

Participating ICUs: Addenbrookes Hospital, John V Farman Intensive Care Unit: Dr Andrew Conway Morris, Dr Matthew Routledge. Addenbrookes Hospital, Neurocritical Care Unit (NCCU): Dr Andrew Conway Morris, Dr Ari Ercole. Charing Cross Hospital - Imperial College NHS Trust, Intensive Care Unit, Level 11: Dr David Antcliffe, Ms Roceld Rojo. Countess Of Chester Foundation Trust, Intensive Care Unit: Dr Kate Tizard, Dr Maria Faulkner. Darlington Memorial Hospital Intensive Care Unit, County Durham and Darlington NHS Foundation Trust: Dr Amanda Cowton, Dr Melanie Kent. Croydon University Hospital, Critical Care Unit: Dr Ashok Raj, Dr Artemis

Zormpa, Dr George Tinaslanidis, Mrs Reena Khade. Department Of Anaesthetics and Intensive Care Medicine, Queen Elizabeth Hospital Birmingham: Dr Tomasz Torlinski, Dr Randeep Mulhi, Dr Shraddha Goyal, Dr Manan Bajaj, Dr Marina Soltan, Dr Aimee Yonan, Dr Rachael Dolan. Department Of Microbiology, Queen Elizabeth Hospital Birmingham: Dr Aimee Johnson. Freeman Hospital, ICCU 37: Dr Caroline Macfie, Dr James Lennard. Hammersmith Hospital - Imperial College NHS Trust, Intensive Care Unit, Level 11: Ms Maie Templeton, Ms Sonia Sousa Arias. James Cook University Hospital, Icu2/3: Dr Uwe Franke, Mr Keith Hugill. Medway Maritime Hospital, Intensive Care Unit: Mrs Hollie Angell. Ninewells Hospital and Medical School NHS Tayside, Intensive Care Unit: Dr Benjamin J Parcell, Dr Katherine Cobb, Dr Stephen Cole. North Cumbria University Hospitals NHS Trust, North Cumbria University Hospitals NHS Trust: Dr Tim Smith, Dr Clive Graham. North Manchester General Hospital, Critical Care Ward: Dr Jaroslav Cerman, Dr Allison Keegan. Queen Elizabeth Hospital, Gateshead Health NHS Foundation Trust, Critical Care Department: Mrs Jenny Ritzema, Mrs Amanda Sanderson. Queen Elizabeth Hospital, Lewisham and Greenwich NHS Trust, Critical Care Unit: Dr Ashraf Roshdy. Royal Gwent Hospital, Critical Care Unit: Dr Tamas Szakmany, Dr Tom Baumer. Royal London Hospital, Adult Critical Care Unit: Dr Rebecca Longbottom, Dr Daniel Hall. Royal Marsden NHS Foundation Trust, Critical Care Unit: Dr Kate Tatham, Dr S Loftus, Dr A Husain, Dr E Black, Dr S Jhanji, Dr R Rao Baikady. Royal Victoria Hospital, Belfast, Regional Intensive Care Unit: Dr Peter Mcguigan, Dr Rachel Mckee. Sandwell And West Birmingham Hospitals NHS Trust, Intensive Care Unit: Dr Santhana Kannan, Dr Supriya Antrolikar, Dr Nicholas Marsden. St Mary's Hospital - Imperial College NHS Trust, Intensive Care Unit, Level 11: Dr Valentina Della Torre, Ms Dorota Banach. Stepping Hill Hospital, Stepping Hill ICU: Dr Ahmed Zaki, Dr Matthew Jackson. University Hospitals of North Midlands, Critical Care Unit: Dr Moses Chikungwa. Warwick Hospital, Intensive Care Unit: Dr Ben Attwood, Dr Jamie Patel. West Suffolk NHS Foundation Trust, Critical Care: Dr Rebecca E Tilley, Miss Sally K Humphreys. Wirral University Teaching Hospital, Intensive Care Unit: Dr Paul Jean Renaud.

Ukraine

Participating ICUs: Kharkiv Clinical Infectious Diseases Hospital, Intensive Care: Prof Anton Sokhan, Dr Yaroslava Burma.

North America

Canada

National Coordinator: Prof. Wendy Sligl

Participating ICUs: University of Alberta Hospital, General Systems Intensive Care Unit (Gsicu): Dr Wendy Sligl, Nadia Baig, Lorena McCoshen. Royal Alexandra Hospital, General Systems Intensive Care Unit (Gsicu): Dr Demetrios J Kutsogiannis, Dr Wendy Sligl, Patricia Thompson, Tayne Hewer.

South Asia

Bangladesh

National Coordinator: Dr Raihan Rabbani

Participating ICUs: General Icu, Dhaka: Dr Raihan Rabbani, Dr Shihan Mahmud Redwanul Huq. Asgar Ali Hospital, Critical Care Medicine: Dr Rajib Hasan, Dr Mohammad Motiul Islam.

India

National Coordinator: Prof. Mohan Gurjar

Participating ICUs: Sanjay Gandhi Postgraduate Institute of Medical Sciences (SGPGIMS), Lucknow, Department of Critical Care Medicine: Dr Mohan Gurjar, Dr Arvind Baronia. All India Institute of Medical Sciences (AIIMS) Jodhpur, Department of Anaesthesiology & Critical Care Medicine: Dr Nikhil Kothari, Dr Ankur Sharma. All India Institute of Medical Sciences (AIIMS), Pulmonary Medicine ICU, Department of Pulmonary Medicine: Dr Saurabh Karmakar, Dr Priya Sharma. Breach Candy Hospital Trust, SICU: Dr Janardan Nimbolkar, Dr Pratit Samdani. Cauvery Heart And Multi-Speciality Hospital, MICU: Dr Vaidyanathan R, Dr Noor Ahmedi Rubina. CHL Hospitals, Dept of Critical Care Services: Dr Nikhilesh Jain, Dr Madhumati Pahuja. Indira Gandhi Institute of Medical Sciences, Trauma & Emergency: Dr Ritu Singh, Dr Saurav Shekhar. King George's Medical University, Department of Critical Care Medicine: Dr Syed Nabeel Muzaffar, Dr Ahmad Ozair, Dr Suhail Sarwar Siddiqui. Medica Superspecialty Hospital, Medica Institute of Critical Care: Dr Payel Bose, Dr Avijatri Datta. Sir H N Reliance Foundation Hospital, Critical Care Unit: Dr Darshana Rathod, Dr Mayur Patel. Sri Ramachandra Institute of Higher Education and Research, Department of Critical Care Medicine: Prof MK Renuka, Dr Sailaja K Baby. St Johns Medical College Hospital, Department of Critical Care Medicine, MICU: Dr Carol Dsilva, Dr Jagadish Chandran. Tata Medical Center, Critical Care Medicine: Dr Pralay Ghosh, Dr Sudipta Mukherjee. Yashoda Hospital, Somajiguda, Hyderabad: Dr Kaladhar Sheshala, Dr Krushna Chandra Misra.

Sub-Saharan Africa

Nigeria

National Coordinator: Dr. Oyebola O. Adekola

Participating ICUs: Ahmadu Bello University Teaching Hospital Shika Zaria Abuth Zari, Icu Abuth Zaria: Dr Saidu Yusuf Yakubu, Dr Euphemia Mgbosoro Ugwu. Lagos University Teaching Hospital, Department of Anaesthesia And Intensive Care: Dr John (O) Olatosi, Dr Ibironke Desalu. One Life Hospital, One Life Intensive Care Unit: Dr Gabriel Asiyanbi, Dr Motunrayo Oladimeji. University College Hospital, Anaesthesia: Dr Olusola Idowu, Dr Fowotade Adeola.

South Africa

National Coordinator: Prof. Mervyn Mer

Participating ICUs: Charlotte Maxeke Johannesburg Academic Hospital, Ward 576: Prof Mervyn Mer, Mrs Melanie Mc Cree.

Sudan

National Coordinator: Dr. Bashir El Sanousi

*Participating ICUs:*Al-Rajhi Hospital, Medicine: Dr Ali Adil Ali Karar. East Nile Hospital, Intensive Care Unit: Dr Elfayadh Saidahmed, Dr Hytham K.S. Hamid.

Participating countries

Supplementary Table S1. Countries reporting data to the EUROBACT-2 study.

Country	Participating centres	Total patients reported	Total patients included in analysis	Patients in EMT group	Patients in ECAT group
Argentina	4 (1.22)	23 (0.96)	16 (0.89)	8 (0.93)	8 (0.84)
Australia	14 (4.27)	98 (4.07)	81 (4.49)	43 (5.01)	38 (4.01)
Bangladesh	2 (0.61)	6 (0.25)	6 (0.33)	2 (0.23)	4 (0.42)
Belgium	12 (3.66)	60 (2.49)	37 (2.05)	24 (2.80)	13 (1.37)
Bosnia and Herzegovina	1 (0.30)	10 (0.42)	8 (0.44)	1 (0.12)	7 (0.74)
Brunei	4 (1.22)	24 (1)	13 (0.72)	12 (1.40)	1 (0.11)
Canada	2 (0.61)	18 (0.75)	15 (0.83)	9 (1.05)	6 (0.63)
China	15 (4.57)	73 (3.03)	52 (2.88)	33 (3.85)	19 (2)
Colombia	1 (0.30)	6 (0.25)	6 (0.33)	5 (0.58)	1 (0.11)
Croatia	2 (0.61)	7 (0.29)	4 (0.22)	1 (0.12)	3 (0.32)
Egypt	7 (2.13)	36 (1.50)	32 (1.77)	23 (2.68)	9 (0.95)
France	34 (10.37)	262 (10.89)	161 (8.91)	79 (9.21)	82 (8.65)
Germany	6 (1.83)	43 (1.79)	32 (1.77)	23 (2.68)	9 (0.95)
Greece	19 (5.79)	138 (5.74)	119 (6.59)	38 (4.43)	81 (8.54)
Hong Kong	1 (0.30)	5 (0.21)	5 (0.28)	4 (0.47)	1 (0.11)
India	11 (3.35)	43 (1.79)	31 (1.72)	17 (1.98)	14 (1.48)
Iran	12 (3.66)	43 (1.79)	39 (2.16)	11 (1.28)	28 (2.95)
Iraq	1 (0.30)	2 (0.08)	1 (0.06)	1 (0.12)	0 (0)
Israel	2 (0.61)	16 (0.67)	16 (0.89)	10 (1.17)	6 (0.63)
Italy	10 (3.05)	146 (6.07)	121 (6.70)	41 (4.78)	80 (8.44)
Japan	11 (3.35)	42 (1.75)	36 (1.99)	16 (1.86)	20 (2.11)
Jordan	1 (0.30)	2 (0.08)	2 (0.11)	0 (0)	2 (0.21)
Kazakhstan	2 (0.61)	7 (0.29)	6 (0.33)	1 (0.12)	5 (0.53)
Lebanon	1 (0.30)	4 (0.17)	2 (0.11)	2 (0.23)	0 (0)
Libya	5 (1.52)	22 (0.91)	15 (0.83)	7 (0.82)	8 (0.84)
Malaysia	6 (1.83)	34 (1.41)	27 (1.50)	26 (3.03)	1 (0.11)
Mexico	6 (1.83)	20 (0.83)	13 (0.72)	3 (0.35)	10 (1.05)
Morocco	6 (1.83)	43 (1.79)	30 (1.66)	8 (0.93)	22 (2.32)
Nigeria	2 (0.61)	5 (0.21)	4 (0.22)	1 (0.12)	3 (0.32)
Philippines	1 (0.30)	8 (0.33)	5 (0.28)	2 (0.23)	3 (0.32)
Poland	6 (1.83)	41 (1.70)	36 (1.99)	23 (2.68)	13 (1.37)
Portugal	13 (3.96)	72 (2.99)	47 (2.60)	31 (3.61)	16 (1.69)
Qatar	3 (0.91)	16 (0.67)	13 (0.72)	2 (0.23)	11 (1.16)
Republic of Ireland	1 (0.30)	8 (0.33)	4 (0.22)	0 (0)	4 (0.42)
Republic of Korea	5 (1.52)	36 (1.50)	31 (1.72)	13 (1.52)	18 (1.90)
Romania	5 (1.52)	36 (1.50)	25 (1.38)	7 (0.82)	18 (1.90)
Russian Federation	5 (1.52)	36 (1.50)	27 (1.50)	20 (2.33)	7 (0.74)
Saudi Arabia	3 (0.91)	14 (0.58)	13 (0.72)	5 (0.58)	8 (0.84)
Singapore	6 (1.83)	18 (0.75)	17 (0.94)	6 (0.70)	11 (1.16)
South Africa	1 (0.30)	5 (0.21)	2 (0.11)	1 (0.12)	1 (0.11)
Spain	12 (3.66)	85 (3.53)	64 (3.54)	24 (2.80)	40 (4.22)
Sudan	2 (0.61)	8 (0.33)	7 (0.39)	5 (0.58)	2 (0.21)
Sweden	3 (0.91)	10 (0.42)	5 (0.28)	5 (0.58)	0 (0)
Switzerland	2 (0.61)	20 (0.83)	18 (1)	10 (1.17)	8 (0.84)
Syria	2 (0.61)	2 (0.08)	2 (0.11)	1 (0.12)	1 (0.11)

Country	Participating centres	Total patients reported	Total patients included in analysis	Patients in EMT group	Patients in ECAT group
Taiwan	4 (1.22)	33 (1.37)	17 (0.94)	8 (0.93)	9 (0.95)
Thailand	2 (0.61)	20 (0.83)	18 (1)	3 (0.35)	15 (1.58)
Tunisia	2 (0.61)	22 (0.91)	14 (0.78)	2 (0.23)	12 (1.27)
Turkey	24 (7.32)	505 (20.99)	384 (21.26)	174 (20.28)	210 (22.15)
Ukraine	1 (0.30)	1 (0.04)	1 (0.06)	0 (0)	1 (0.11)
United Arab Emirates (Dubai)	1 (0.30)	10 (0.42)	5 (0.28)	1 (0.12)	4 (0.42)
United Kingdom	24 (7.32)	162 (6.73)	121 (6.70)	66 (7.69)	55 (5.80)

ECAT - empiric combination antibiotic therapy; EMT - empiric antibiotic monotherapy.

Definitions

The EUROBACT-2 study included adult (≥ 18 years old) patients with a HABSI treated in the ICU. [1] HA-BSI was defined as a positive blood culture sample collected more than 48 h after hospital admission. [1] Sepsis-3 definitions for sepsis and septic shock were used. [1,2]

The process of patient classification into ECAT and EMT groups is illustrated in Supplementary Figure 1. Antibiotic therapy was classified as empiric based upon the following criteria:

- The primary indication for initiation of treatment was reported as empiric therapy for HA-BSI (i.e. not targeted therapy, de-escalation on the basis of antibiotic susceptibility testing, or treatment of other infections) by physicians participating in EUROBACT-2.
- Treatment was either initiated within 48 hours after initial blood culture sampling, or, if treatment was started before initial blood culture sampling, it had to have continued during the first 48 hours thereafter. For example, empiric treatment initiated three days after the initial blood cultures were sampled, was not included in the analysis. The same applied for empiric antibiotic therapy initiated and terminated before initial blood culture sampling.

ECAT was defined as empiric therapy for HA-BSI using two or more antibiotic agents of different classes (e.g., beta-lactam plus aminoglycoside). Treatment with two or more agents of the same antibiotics class (e.g. two aminoglycoside agents) was not classified as ECAT. If patients were treated for multiple infections with a broad combination of antibiotics, only those indicated as being intended as empiric treatment for HA-BSI were included in our definition (i.e., if a patient was treated with five antibiotic agents simultaneously yet only two were indicated as empiric therapy for HA-BSI, only the two agents were considered as ECAT).

EMT was defined as empiric therapy for HA-BSI using a single antibiotic agent. Patients receiving multiple antibiotic agents simultaneously where only one agent was specified as having been used for empiric treatment of HA-BSI were also classified in the EMT group. For instance, a patient who received ceftriaxone for empiric HA-BSI treatment while simultaneously receiving vancomycin as part of prior targeted therapy was included in the EMT group.

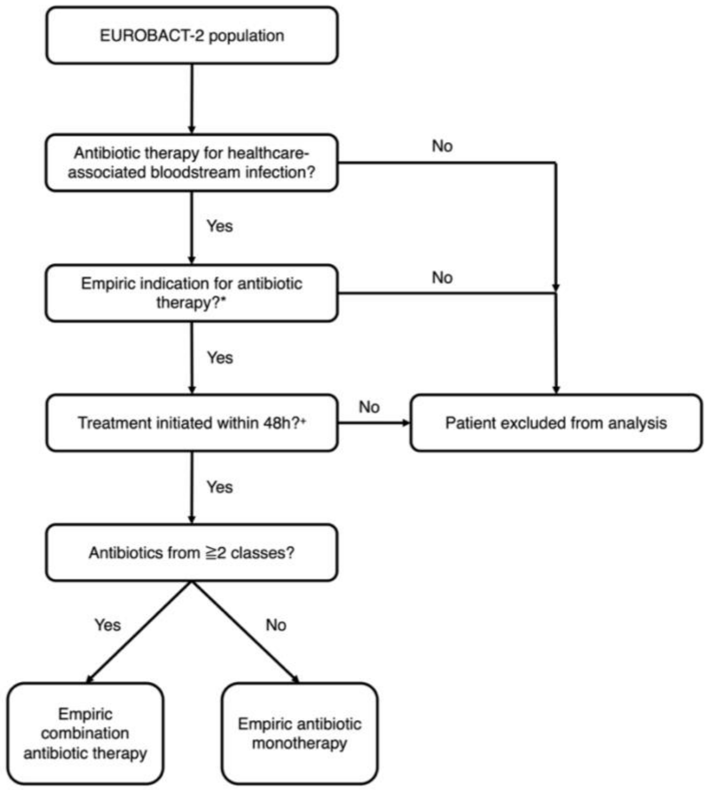

* - as indicated by reporting physicians participating in the EUROBACT-2 study. $^{+}$ - treatment initiated within 48 hours from initial blood culture sampling for healthcare-associated bloodstream infections or treatment initiated prior to blood culture sampling and continued during the first 48 hours after sampling.

Supplementary Figure 1. Flowchart illustrating the criteria used for patient inclusion and stratification.

Immune deficiency was defined as the presence of any of the following: malignant tumours regardless of the presence of metastases; haematological malignancy (lymphoma or leukaemia); other solid tumours; presence of transplanted organs; treatment with high-dose steroids; other immunosuppression. The frequencies of immune deficiency causes in the study population are outlined in Supplementary Table S3.

Supplementary Table S2. Frequencies of immune deficiency causes.

Cause of immune deficiency	EMT, n (%)	ECAT, n (%)	Total, n (%)
Active malignancy, no metastasis	82 (9.5)	97 (10.2)	179 (9.9)
Metastasised malignancy	50 (5.8)	72 (7.6)	122 (6.7)
Haematological malignancy	45 (4.9)	84 (8.9)	129 (7.1)
Solid tumour, other	22 (2.6)	35 (3.7)	57 (3.1)
Organ transplant	20 (2.3)	27 (2.8)	47 (2.6)
High dose steroids	34 (4.0)	65 (6.8)	99 (5.5)
Other immunosuppression	39 (4.5)	36 (3.8)	75 (4.1)

ECAT - empiric combination antibiotic therapy; EMT - empiric antibiotic monotherapy. Data was stratified by treatment group and indicated as percentage of the final analysis population. The numbers of instances of diagnoses are shown, meaning that the same patient may have been represented in several categories.

Statistical analysis

Patients were stratified into groups based on whether they received EMT or ECAT and descriptive statistics tables were generated. Continuous variables were presented as median values with corresponding interquartile ranges. Categorical values were presented as total counts and percentages.

A multilevel logistic regression approach was employed, with individual patients (level 1) were nested within ICUs (level 2), which were further nested within countries (level 3). This clustering leads to statistical correlation and reduces the effective sample size. [3] Multilevel modelling accounts for this intraclass correlation, ensuring accurate estimation of uncertainty (e.g., 95% confidence intervals) around measures of association (e.g., odds ratios). From a clinical perspective, the existence of patient correlation within ICUs and countries is valuable and reflects practice styles or therapeutic traditions. [4] For instance, the same patient might receive different treatments in different ICUs. Thus, multilevel modelling is particularly well-suited to address the aims of our study. In addition, the hierarchical structure allows for the investigation of both patient- and ICU-level factors influencing treatment decisions, and for the calculation of variance partition coefficients, which indicate the total impact of institutional and national variables, including potential factors that may not have been included in the model. [3]

In the regression model, patient and ICU related variables were modelled as fixed effects, with specific ICUs and countries used as random effects. Continuous variables were centred around their respective grand population means where appropriate. If variables were not centred, the intercept would represent the log-odds of the outcome for a patient with a BMI of 0, which is not a meaningful reference point. Centring the values, on the other hand, allows for interpretation of intercept values, as the "average" patient will be represented. This is, however, not applicable to all continuous variables, e.g. the Sequential organ failure (SOFA) score is equal to 0 in the healthy person. To lower the total number of parameters in the regression model, ordinal variables containing large numbers of categories were contracted to include fewer, broader categories when deemed necessary.

Clinically relevant patient-level variables and institutional factors such as the type of ICU (mixed, medical-only, or surgical-only), hospital academic status (teaching vs. non-teaching), and availability of national or international treatment guidelines were added to the model:

$$
\begin{aligned}
\text{combination\_therapy}_{ijk} \quad & \sim \text{Binomial}\left(\text{cons}_{ijk}, \pi_{ijk}\right) \\
\text{logit}\left(\pi_{ijk}\right) \quad & = \beta_{0jk}\text{cons} + \beta_1(\text{Age})_{ijk} + \beta_2\text{Female sex}_{ijk} + \beta_3(\text{BMI})_{ijk} \\
& \quad +\beta_4\text{Immune deficiency}_{ijk} + \beta_5\text{Medical admission}_{ijk} \\
& \quad +\beta_6\text{Surgical emergency admission}_{ijk} + \beta_7\text{Early ICU-acquired infection (<7 days)}_{ijk} \\
& \quad +\beta_8\text{Late ICU-acquired infection (>7 days)}_{ijk} + \beta_9\text{SOFA 8-11}_{ijk} \\
& \quad +\beta_{10}\text{SOFA > 11}_{ijk} + \beta_{11}\text{Septic shock}_{ijk} + \beta_{12}\text{Primary infection}_{ijk} \\
& \quad +\beta_{13}\text{Catheter infection}_{ijk} + \beta_{14}\text{Intra-abdominal infection}_{ijk} \\
& \quad +\beta_{15}\text{Urinary infection}_{ijk} + \beta_{16}\text{Other infection}_{ijk} \\
& \quad +\beta_{17}\text{Use of national guidelines}_{jk} + \beta_{18}\text{Non-teaching Hospital}_{jk} \\
& \quad +\beta_{19}\text{Medical ICU}_{jk} + \beta_{20}\text{Surgical ICU}_{jk} \\
& \quad +\beta_{21}\text{Infectious diseases specialist available 24/7}_{jk} \\
& \quad +\beta_{22}\text{Clinical pharmacist available 24/7}_{jk} + \beta_{23}\text{MRSA 10-25\%}_{jk} \\
& \quad +\beta_{24}\text{MRSA > 25\%}_{jk} + \beta_{25}\text{MRSA unknown}_{jk} + \beta_{26}\text{VRE 10-25\%}_{jk} \\
& \quad +\beta_{27}\text{VRE > 25\%}_{jk} + \beta_{28}\text{VRE unknown}_{jk} + \beta_{29}\text{ESBL 10-25\%}_{jk} \\
& \quad +\beta_{30}\text{ESBL > 25\%}_{jk} + \beta_{31}\text{ESBL unknown}_{jk} + \beta_{32}\text{EC 10-25\%}_{jk} \\
& \quad +\beta_{33}\text{EC > 25\%}_{jk} + \beta_{34}\text{EC unknown}_{jk} \\
\beta_{0jk} \quad & = \beta_0 + \nu_{0jk} + u_{0jk} \\
[\nu_{0jk}] \quad & \sim \text{N}(0, \Omega_\nu) : \Omega_\nu = \left[\sigma^2_{\nu_0}\right] \\
[\nu_{0jk}] \quad & \sim \text{N}(0, \Omega_u) : \Omega_u = \left[\sigma^2_{u_0}\right]
\end{aligned}
$$

Where:

- $\text{combination\_therapy}_{ijk}$: binary outcome indicating combination therapy for individual i in ICU j within country k.
- cons_{ijk} - constant value (1).
- π_{ijk}: probability of combination therapy for individual i in ICU j within country k.
- $\text{logit}\left(\pi_{ijk}\right)$: log-odds of combination therapy.
- β_{0jk}: group-level intercept for ICU j within country k.
- $\beta_1, \beta_2, \dots, \beta_{34}$: fixed effect coefficients for covariates (e.g., age, sex, ICU characteristics).
- ν_{0jk}: random intercept at the ICU level (level 2) with variance $\sigma^2_{\nu_0}$.
- u_{0jk}: random intercept at the country level (level 3) with variance $\sigma^2_{u_0}$.
- Ω_ν: variance at the ICU level (level 2), where $\Omega_\nu = \sigma^2_{\nu_0}$.
- Ω_u: variance at the country level (level 3), where $\Omega_u = \sigma^2_{u_0}$.
- $\text{N}(0, \Omega)$: Normal distribution with mean 0 and variance Ω.

The reference (intercept) values used in the model were: age and BMI centred around the grand mean; male sex; admission for elective surgery; no immune deficiency; primary respiratory infection; hospital-acquired infection (i.e. not acquired in the ICU); SOFA-score < 8 on day one of HA-BSI; no septic shock on HA-BSI presentation; treatment in an academic hospital; infectious disease specialist/clinical microbiologist/clinical pharmacist consultations not available 24/7; proportions of multidrug-resistant isolates < 10%.

Markov-chain Monte Carlo (MCMC) estimation based on the Metropolis-Hastings algorithm was used to assess the effects of patient related factors on the odds of patients receiving ECAT. Non-informative priors were used. Logistic regression using the iterative generalised least squares (IGLS) procedure was performed to generate the starting values for MCMC estimation. A dichotomous yes/no outcome variable and the logit-link function, allowing for calculation of odds ratios by exponentiation of regression coefficients, were used. Regression results are presented as regression coefficients with standard errors and odds ratios with corresponding 95% credible intervals.

The impact of institutional and national factors, such as therapeutic traditions, availability of monitoring or interventions, or local epidemiological factors, on the variations in the likelihood for patients to receive ECAT, was evaluated (Table 4). This was done by calculating the intra-class correlation coefficient (ICC), which measures the proportion of total variance attributable to higher-level clustering (e.g., ICUs or countries). [3]

First, a variance component model (Model 0) including only the random intercepts was created:

$$\begin{aligned}
\text{combination_therapy}_{ijk} &\sim \text{Binomial}\left(\text{cons}_{ijk}, \pi_{ijk}\right) \\
\text{logit}\left(\pi_{ijk}\right) &= \beta_{0jk}\text{cons} + \nu_{0jk} + u_{0jk} \\
[\nu_{0jk}] &\sim \text{N}(0, \Omega_\nu) : \Omega_\nu = \left[\sigma^2_{\nu_0}\right] \\
[\nu_{0jk}] &\sim \text{N}(0, \Omega_u) : \Omega_u = \left[\sigma^2_{u_0}\right]
\end{aligned}$$

Where:

- $\text{combination\_therapy}_{ijk}$ - binary outcome indicating combination therapy for individual i in ICU j within country k.
- cons_{ijk} - constant value (1).
- π_{ijk}: probability of combination therapy for individual i in ICU j within country k.
- $\text{logit}\left(\pi_{ijk}\right)$: log-odds of combination therapy.

- β_{0jk}: group-level intercept for ICU j within country k.
- v_{0jk}: random intercept at the ICU level (level 2) with variance $\sigma^2_{v_0}$.
- u_{0jk}: random intercept at the country level (level 3) with variance $\sigma^2_{u_0}$.
- Ω_v: variance at the ICU level (level 2), where $\Omega_v = \sigma^2_{v_0}$.
- Ω_u: variance at the country level (level 3), where $\Omega_u = \sigma^2_{u_0}$.
- $\mathrm{N}(0, \Omega)$: Normal distribution with mean 0 and variance Ω.

ICC was then calculated as follows:

$$\mathrm{ICC}_{ICU} = \frac{\Omega_v + \Omega_u}{\Omega_v + \Omega_u + 3.29}$$

$$\mathrm{ICC}_{Country} = \frac{\Omega_u}{\Omega_v + \Omega_u + 3.29}$$

Where:

- $\mathrm{ICC}_{\Omega v}$: intra-class correlation coefficient.
- Ω_v : represents the ICU-level (level 2) variance.
- Ω_u : represents the country-level (level 3) variance.
- 3.29 : is the variance of a standard logistic distribution ($\pi^2/3$) representing patient-level variance in a logistic regression model.

Expressed as a percentage, the ICC ranges from 0% to 100%. Higher values indicate that a greater proportion of the variation in ECAT prescription is explained by differences between ICUs and countries, such as therapeutic traditions or treatment accessibility.

As seen previously, the variance component model includes only the random effects, with no fixed effects. Feeding the regression model additional information by introducing additional variables should decrease the result variance, thus lowering the effects of clustering seen in the variance component model. We examined this scenario by creating multilevel logistic regression models including both fixed and random effects and calculating ICC and proportional change of variance (PCV) in a stepwise fashion. Risk scores (predicted probabilities) for the use of ECAT were

calculated for each patient in a single-level logistic regression models and then divided into quartiles. Using risk scores instead of individual variables accounted for individual variations in a more parsimonious fashion and simplified multivariable adjustment. The initial model (Model 1) used for calculating risk scores included patient-level variables only. Risk scores estimated using the second model (Model 2) included both patient and ICU-level variables. A multilevel logistic regression model using risk score quartiles as fixed effects, as well as ICU and country as random effects, was then created, and MCMC was used to estimate the result variance:

$$
\begin{aligned}
\text{combination\_therapy}_{ijk} &\sim \text{Binomial}\left(\text{cons}_{ijk}, \pi_{ijk}\right) \\
\text{logit}\left(\pi_{ijk}\right) &= \beta_{0jk}\text{cons} + \beta_1(\text{Q2})_{ijk} + \beta_2(\text{Q3})_{ijk} + \beta_3(\text{Q4})_{ijk} \\
\beta_{0jk} &= \beta_0 + \nu_{0jk} + u_{0jk} \\
[\nu_{0jk}] &\sim \text{N}(0, \Omega_\nu) : \Omega_\nu = \left[\sigma^2_{\nu_0}\right] \\
[\nu_{0jk}] &\sim \text{N}(0, \Omega_u) : \Omega_u = \left[\sigma^2_{u_0}\right]
\end{aligned}
$$

Where:

- $\text{combination\_therapy}_{ijk}$: binary outcome indicating combination therapy for individual i in ICU j within country k.
- cons_{ijk} - constant value (1).
- π_{ijk}: probability of combination therapy for individual i in ICU j within country k.
- $\text{logit}\left(\pi_{ijk}\right)$: log-odds of combination therapy.
- β_{0jk}: group-level intercept for ICU j within country k.
- $\beta_1, \beta_2, \beta_3$: fixed effect coefficients for covariates.
- Q2, Q3, Q4 - quartiles of the risk score for ECAT.
- ν_{0jk}: random intercept at the ICU level (level 2) with variance $\sigma^2_{\nu_0}$.
- u_{0jk}: random intercept at the country level (level 3) with variance $\sigma^2_{u_0}$.
- Ω_ν: variance at the ICU level (level 2), where $\Omega_\nu = \sigma^2_{\nu_0}$.
- Ω_u: variance at the country level (level 3), where $\Omega_u = \sigma^2_{u_0}$.
- $\text{N}(0, \Omega)$: Normal distribution with mean 0 and variance Ω.

Variance values from the two models were extracted for both the ICU and country levels. In addition to calculating the ICC for each model, the models were compared by calculating the PCV as follows:

$$PCV = 100 \times \frac{\Omega_{\text{Model 1}} - \Omega_{\text{Model 2}}}{\Omega_{\text{Model 1}}}$$

Overall, this approach helped distinguish whether variation in ECAT use was primarily due to differences in patient cohorts across ICUs and countries or to institutional and national factors such as therapeutic traditions. [5]

Empiric combination antibiotic therapy stratified by exposure

Supplementary Table S3. Antibiotic combinations used for empiric combination antibiotic therapy stratified by select exposures.

	Immune deficiency	Uncommon infection Sources	SOFA >11	CPE >25%
**Combination**	**n (% ECAT) [% Total]**	**n (% ECAT) [% Total]**	**n (% ECAT) [% Total]**	**n (% ECAT) [% Total]**
**Gram-negative coverage**				
Aminoglycoside + Beta-lactam	76 (8) [4.20]	11 (1.16) [0.61]	51 (5.37) [2.82]	22 (2.32) [1.22]
Beta-lactam + Fluoroquinolone	34 (3.58) [1.88]	8 (0.84) [0.44]	26 (2.74) [1.44]	17 (1.79) [0.94]
Beta-lactam + Colistin	43 (4.53) [2.38]	8 (0.84) [0.44]	47 (4.95) [2.60]	86 (9.05) [4.75]
**Anaerobe coverage**				
Beta-lactam + Metronidazole	29 (3.05) [1.60]	14 (1.47) [0.77]	19 (2) [1.05]	15 (1.58) [0.83]
Carbapenem + Metronidazole	6 (0.63) [0.33]	2 (0.21) [0.11]	6 (0.63) [0.33]	7 (0.74) [0.39]
Metronidazole + Piperacillin/Tazobactam	5 (0.53) [0.28]	2 (0.21) [0.11]	4 (0.42) [0.22]	4 (0.42) [0.22]
Lincosamide + Metronidazole	0 (0) [0]	2 (0.21) [0.11]	1 (0.11) [0.06]	1 (0.11) [0.06]
**Gram-positive coverage**				
Beta-lactam + Glycopeptide	135 (14.21) [7.46]	36 (3.79) [1.99]	130 (13.68) [7.18]	103 (10.84) [5.69]
Beta-lactam + Lincosamide	8 (0.84) [0.44]	5 (0.53) [0.28]	5 (0.53) [0.28]	5 (0.53) [0.28]
Beta-lactam + Oxazolidinone	38 (4) [2.10]	13 (1.37) [0.72]	40 (4.21) [2.21]	48 (5.05) [2.65]
**Coverage for atypical pathogens**				
Beta-lactam + Macrolide	5 (0.53) [0.28]	2 (0.21) [0.11]	9 (0.95) [0.50]	1 (0.11) [0.06]

ECAT - empiric combination antibiotic therapy; SOFA - sequential Organ Failure Score; CPE - proportion of carbapenemase producing *Enterobacteriaceae* isolates in the intensive care unit; * - proportion of total patient population included in the final analysis (n = 1810).

Sensitivity analysis results

Supplementary Table S4. Characteristics of patients receiving empiric combination antibiotic therapy or empiric antibiotic monotherapy exclusively for the treatment of hospital-associated bloodstream infections.

**Variable**		**EMT**	**ECAT**
Number of patients, n		177	287
Age (median [IQR])		67.0 [55.0–76.0]	65.0 [54.0–76.0]
Sex, n (%)	Male	103 (58.2)	191 (66.6)
	Female	74 (41.8)	96 (33.4)
BMI (median [IQR])		26.4 [23.5–29.3]	26.1 [23.4–29.4]
Charlson comorbidity index, n (%)	0	57 (32.2)	82 (28.6)
	1-2	62 (35.0)	101 (35.2)
	>2	58 (32.8)	104 (36.2)
Immune deficiency†, n (%)		34 (19.2)	88 (30.7)
Cause of ICU-admission, n (%)	Medical	127 (71.8)	218 (76.0)
	Surgical elective	15 (8.5)	14 (4.9)
	Surgical emergency	35 (19.8)	55 (19.2)
Modified SAPS 2* (median [IQR])		35.0 [26.0–49.0]	37.0 [26.5–51.0]
Timing of HA-BSI, n (%)	Hospital-acquired	29 (16.4)	68 (23.7)
	Early ICU-acquired (≤7 days)	74 (41.8)	82 (28.6)
	Late ICU-acquired (>7 days)	74 (41.8)	137 (47.7)
Likely primary source of infection, n (%)	Primary	28 (15.8)	42 (14.6)
	Catheter	38 (21.5)	84 (29.3)
	Intra-abdominal	30 (16.9)	44 (15.3)
	Respiratory	55 (31.1)	77 (26.8)
	Urinary	13 (7.3)	20 (7.0)
	Other	13 (7.3)	20 (7.0)
SOFA score‡, n (%)	< 8	67 (37.9)	89 (31.0)
	8-11	63 (35.6)	88 (30.7)
	>11	47 (26.6)	110 (38.3)
Sepsis‡, n (%)		173 (97.7)	283 (98.6)
Septic shock‡, n (%)		73 (41.2)	129 (44.9)
Vasopressor use‡, n (%)		101 (57.1)	193 (67.2)
Respiratory support‡, n (%)	Low flow Oxygen or no oxygen	33 (18.6)	48 (16.7)
	High Flow Oxygen Nasal Canula	15 (8.5)	21 (7.3)
	Non-Invasive Mechanical Ventilation or CPAP	15 (8.5)	17 (5.9)
	Invasive Mechanical Ventilation	114 (64.4)	201 (70.0)
Renal replacement therapy‡, n (%)	Yes	31 (17.5)	61 (21.3)
ECMO‡, n (%)	Yes	1 (0.6)	6 (2.1)

BMI - body mass index; CPAP - continuous positive airway pressure; ECAT - empiric combination antibiotic therapy; ECMO - extracorporeal membrane oxygenation; EMT - empiric antibiotic monotherapy; HA-BSI – hospital-associated bloodstream infection; ICU - intensive care unit; SAPS 2 - simplified acute physiology scale; SOFA - sequential organ failure assessment; * - simplified acute physiology score II upon admission, age variable subtracted; † - prior to debut of HA-BSI; ‡ - on the day of initial blood culture sampling.

Supplementary Figure 2. Multilevel logistic regression estimations of variable effects on the odds of EMT and ECAT use in the sensitivity analysis population.

Variables	EMT (n = 177)	ECAT (n = 287)	Odds ratio (95% CrI)
Patient variables			
Age	67.0 [55.0–76.0]	65.0 [54.0–76.0]	1.00 (0.98 to 1.01)
Female sex	74	96	0.73 (0.41 to 1.29)
BMI	26.4 [23.5–29.3]	26.1 [23.4–29.4]	1.02 (0.98 to 1.07)
Immune deficiency	34	88	2.32 (1.15 to 4.67)
Admission cause			
Medical	127	218	1.85 (0.58 to 5.91)
Surgical emergency	35	55	2.73 (0.77 to 9.73)
Timing of HA–BSI			
Early ICU–acquired (<=7 days)	74	82	0.43 (0.19 to 0.99)
Late ICU–acquired (>7 days)	74	137	0.70 (0.30 to 1.64)
Severity of illness			
SOFA score 8–11	63	88	1.42 (0.72 to 2.80)
SOFA score >11	47	110	2.07 (0.94 to 4.56)
Septic shock	73	129	0.95 (0.48 to 1.86)
Most likely source of infection			
Primary	28	42	1.41 (0.58 to 3.38)
Catheter	38	84	2.25 (1.00 to 5.09)
Intra–abdominal	30	44	0.77 (0.29 to 2.07)
Urinary	13	20	1.45 (0.46 to 4.58)
Other	13	20	1.69 (0.49 to 5.78)
Institutional variables			
Antibiotic therapy based on national guidelines	104	154	0.91 (0.40 to 2.04)
Non–teaching Hospital	21	27	1.04 (0.29 to 3.73)
Type of ICU			
Medical	22	34	0.92 (0.29 to 2.96)
Surgical	5	10	1.48 (0.18 to 11.95)
Availability of specialist consultation			
Infectious disease specialist available 24/7	86	171	1.47 (0.63 to 3.41)
Clinical pharmacist available 24/7	42	74	1.11 (0.42 to 2.90)
Proportion of MRSA in ICU*			
10–25%	48	56	0.95 (0.33 to 2.71)
>25%	36	79	1.83 (0.58 to 5.78)
Unknown	15	40	0.98 (0.07 to 14.37)
Proportion of VRE in ICU*			
10 – 25%	34	41	0.45 (0.13 to 1.56)
>25%	6	12	1.91 (0.30 to 12.18)
Unknown	18	46	1.18 (0.10 to 14.06)
Proportion of ESBL in ICU*			
10–25%	31	52	0.85 (0.27 to 2.70)
>25%	71	115	0.91 (0.28 to 2.92)
Unknown	23	54	1.44 (0.17 to 12.25)
Proportion of CPE in ICU*			
10–25%	48	39	0.30 (0.09 to 1.03)
>25%	33	86	0.92 (0.27 to 3.18)
Unknown	21	46	0.65 (0.07 to 6.07)

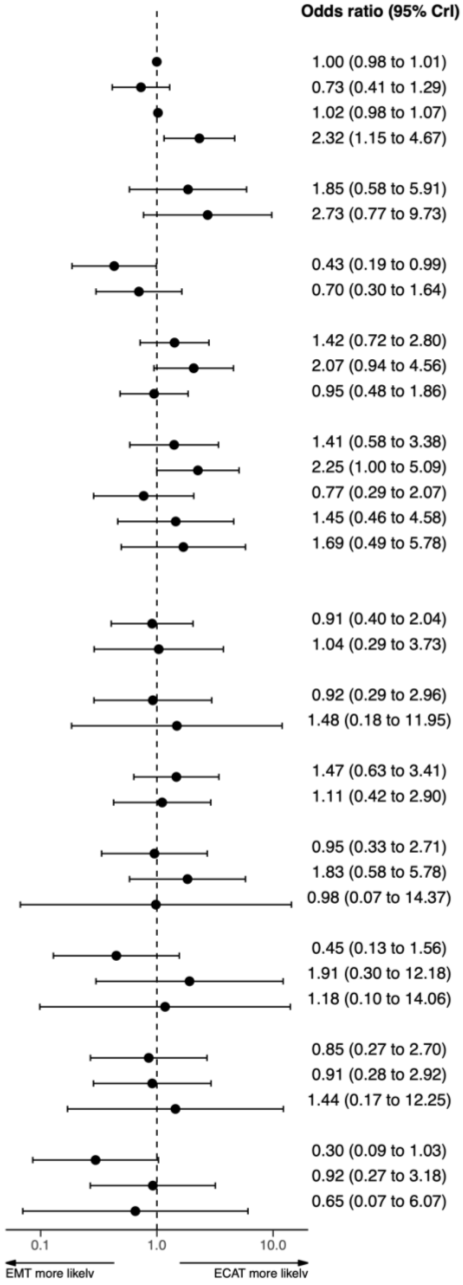

BMI - body mass index; CPE - Carbapenemase producing Enterobacteriaceae; ECAT – empiric combination antibiotic therapy; EMT – empiric antibiotic monotherapy; ESBL - Enterobacteriaceae producing extended-spectrum ß-lactamases; HA-BSI - hospital-associated blood-stream infection; ICU - intensive care unit; MRSA - Staphylococcus aureus resistant to methicillin; SOFA - Sequential Organ Failure Assessment; VRE - Enterococcus spp. resistant to vancomycin; 95% CrI – 95% credible interval; * - percentage of bacterial species isolates within the reporting ICU resistant to the indicated antimicrobial. Patient counts are shown for categorical variables. Median values with interquartile ranges are shown for continuous variables.

Testing Model Fit

Ranked residual plots were generated for levels 2 and 3 (ICU and country, respectively) in the multilevel regression model (Supplementary Figures 3-4). Deviations from a horizontal line are visible in the plots, indicating that the addition of more contextual variables reflecting the significant disparities in factors such as levels of economic development, medical traditions, and antibiotic resistance patterns, between intensive care units (level 2) and countries (level 3) participating in EUROBACT-2, may improve model fit. Therefore, while the model used in this study indicates general trends and associations, these results should be interpreted with caution. Further analyses with larger datasets including additional variables are warranted.

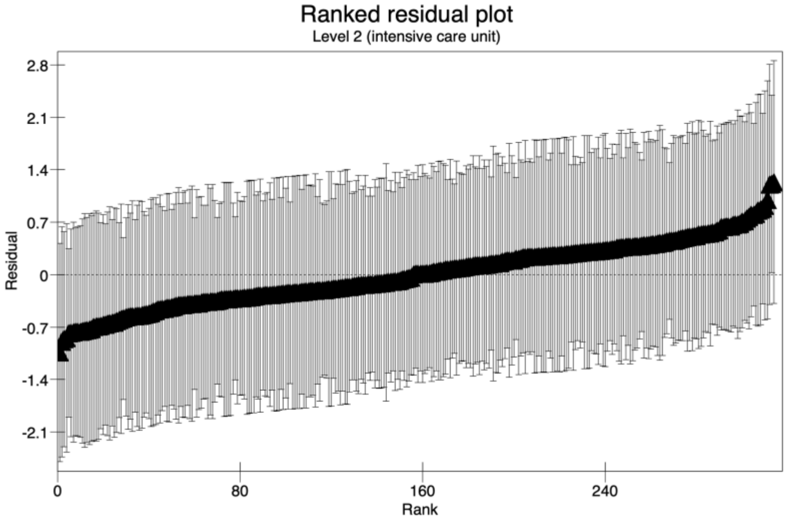

Supplementary Figure 3. Ranked residual plot for level 2 in the multilevel regression model.

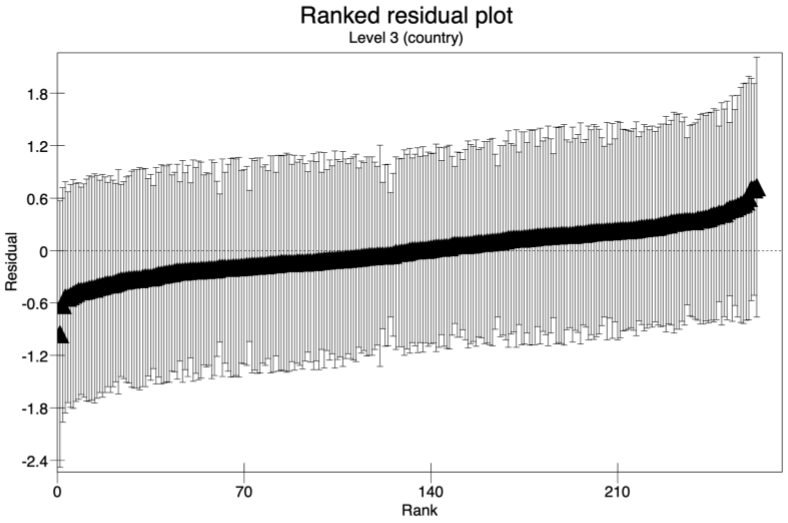

Supplementary Figure 4. Ranked residual plot for level 3 in the multilevel regression model.

References

1. Tabah A, Buetti N, Staiquly Q, Ruckly S, Akova M, Aslan AT, et al. Epidemiology and outcomes of hospital-acquired bloodstream infections in intensive care unit patients: the EUROBACT-2 international cohort study. Intensive Care Med. 2023;49:178–90.

2. Singer M, Deutschman CS, Seymour CW, Shankar-Hari M, Annane D, Bauer M, et al. The Third International Consensus Definitions for Sepsis and Septic Shock (Sepsis-3). JAMA. 2016;315:801–10.

3. Merlo J, Chaix B, Ohlsson H, Beckman A, Johnell K, Hjerpe P, et al. A brief conceptual tutorial of multilevel analysis in social epidemiology: using measures of clustering in multilevel logistic regression to investigate contextual phenomena. J Epidemiol Community Health. 2006;60:290–7.

4. Ohlsson H, Merlo J. Understanding the effects of a decentralized budget on physicians' compliance with guidelines for statin prescription--a multilevel methodological approach. BMC Health Serv Res. 2007;7:68.

5. Merlo J. A brief conceptual tutorial on multilevel analysis in social epidemiology: investigating contextual phenomena in different groups of people. J Epidemiol Community Health. 2005;59:729–36.
